# Supplementary figures and images for: Application of sequential cyclic compression on cancer cells in a flexible microdevice
Source: PLoS One. 2023 Jan 5;18(1):e0279896. doi: 10.1371/journal.pone.0279896 (PMC9815655; doi:10.1371/journal.pone.0279896)

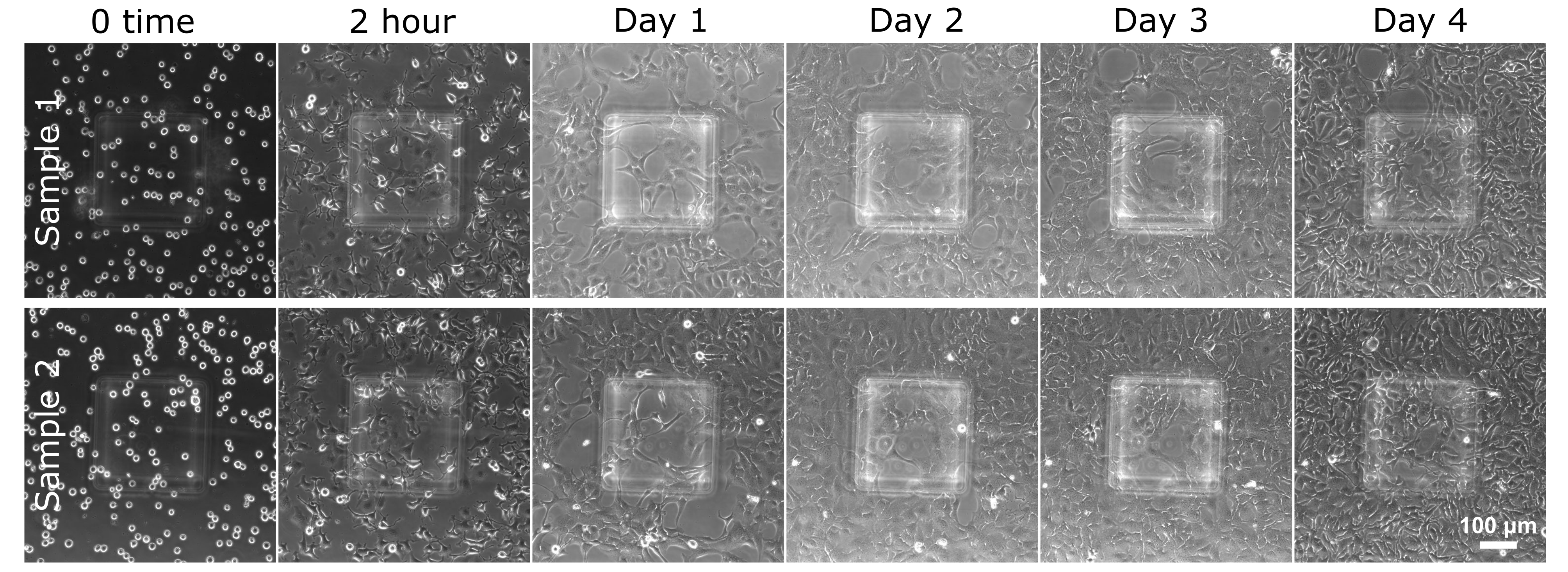

Supplement: S1 Fig — Representative images of two samples during culture from zero time to Day 4 after piston-retracted loading of cells at -615 mbar. (TIF) [file pone.0279896.s001.tif]

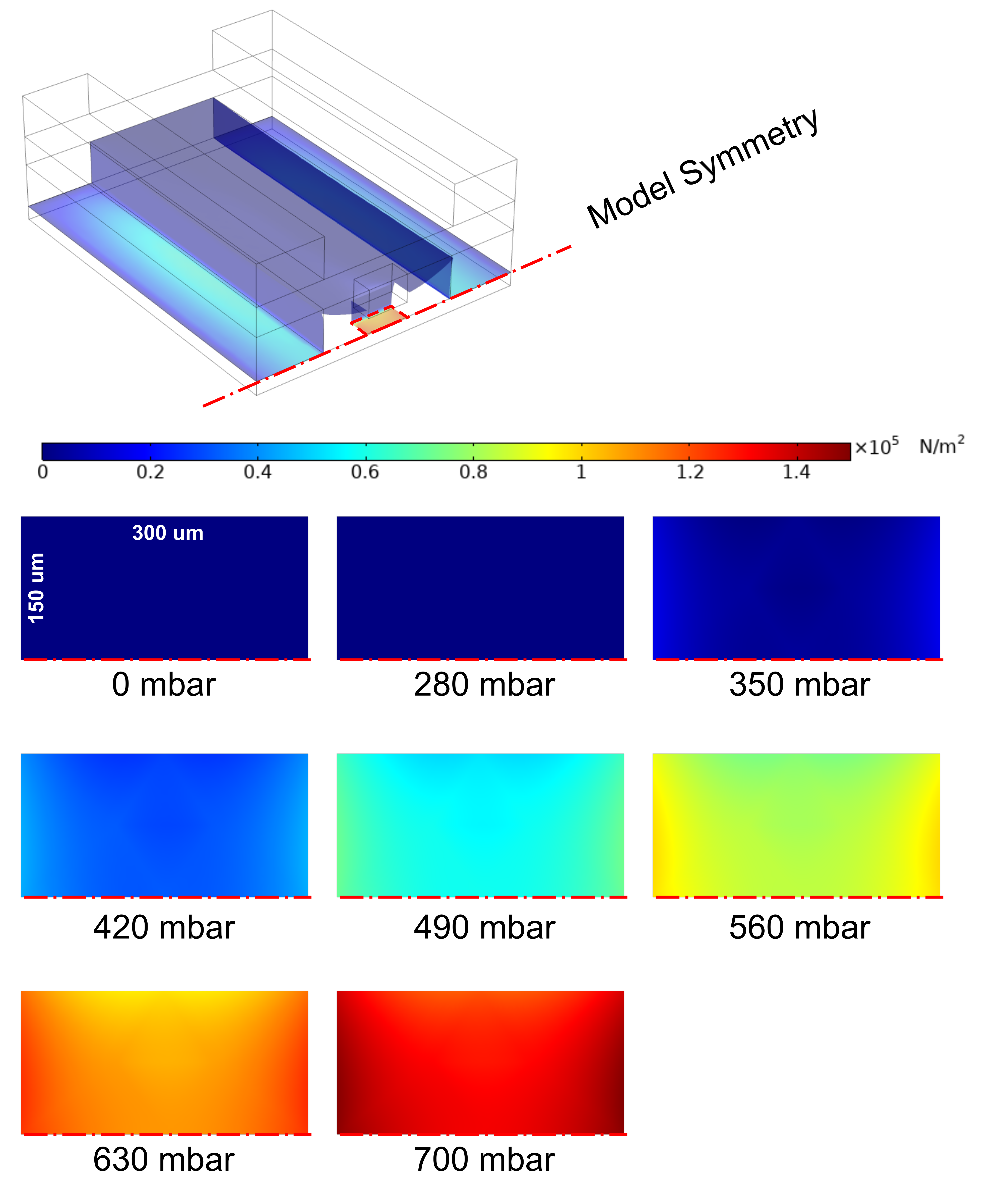

Supplement: S2 Fig — They show the distribution of contact pressure (in N/m2) under piston for the selected applied external pressures (in mbar). Red dashed line of model symmetry drawn on the illustrations at different pressures point out the middle side of the piston. The contact pressure is slightly higher at the edges of the piston towards the side channel walls where the attached membrane distance between piston and side wall is shorter. Thus, there seem to be some variation due to how the piston bottom gets squashed in these sides of the piston, but it is minimal compared to the overall area. This is demonstrated by the color being relatively even over the whole area, especially for externally applied lower pressures such as 0–420 mbar. (TIF) [file pone.0279896.s002.tif]

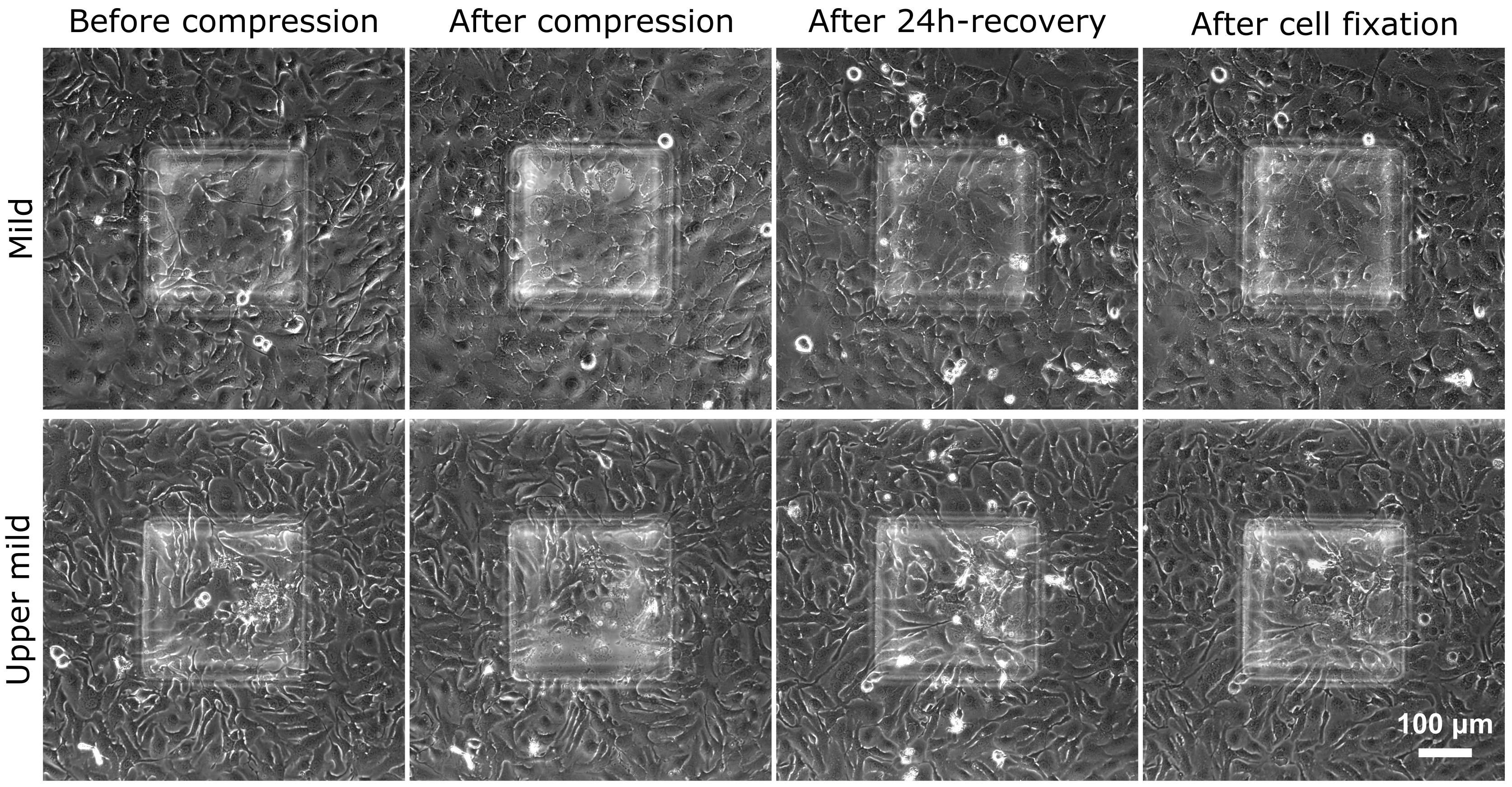

Supplement: S3 Fig — (TIF) [file pone.0279896.s003.tif]

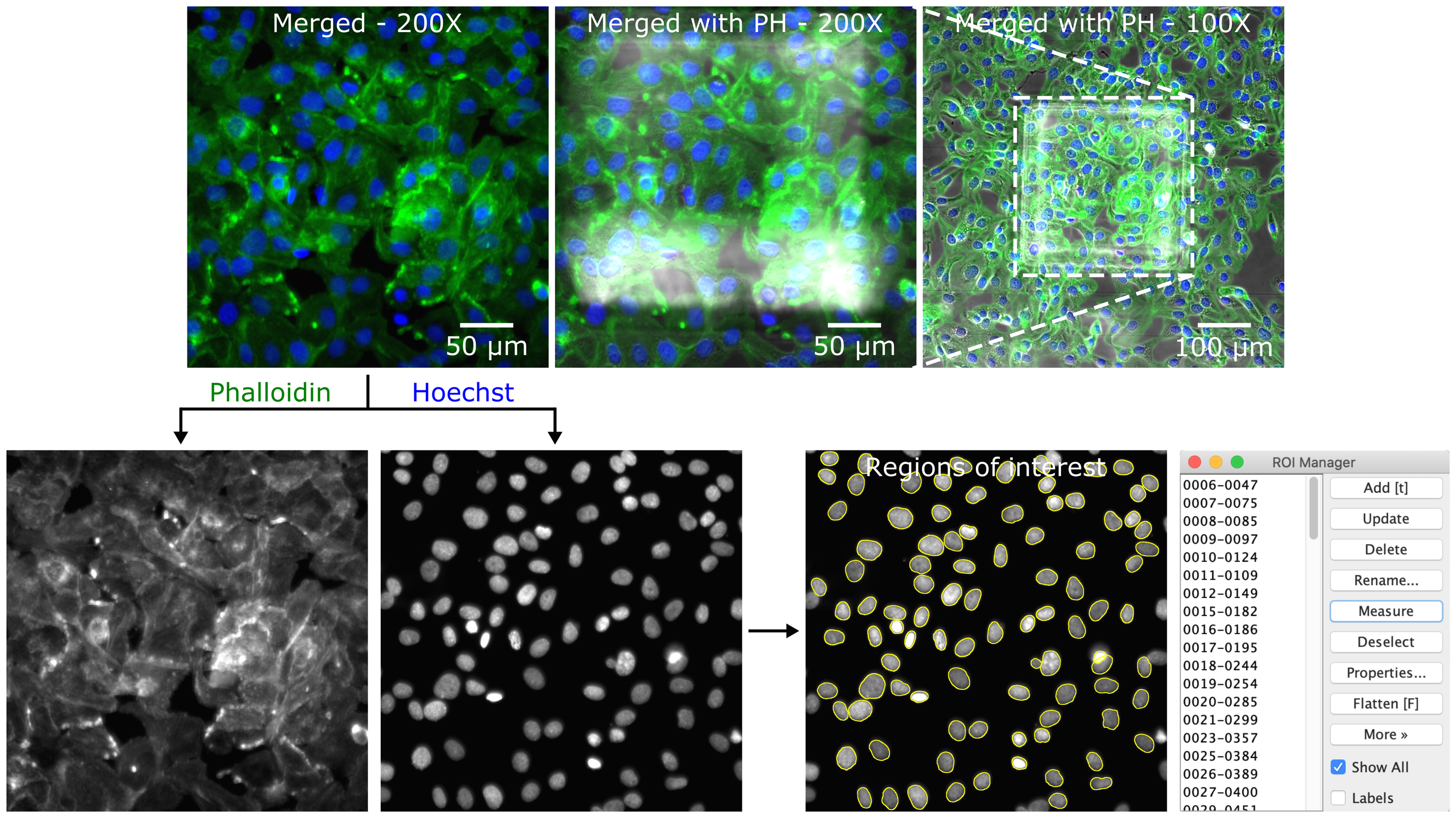

Supplement: S4 Fig — Regions of interest (ROIs) extracted for the cell nuclear boundaries in Hoechst (stain for nuclei) epi-fluorescence channel (blue in merged/composite frames) were used to measure area, circularity, and aspect ratio of the cell nuclei, as independent of phalloidin (stain for actin) epi-fluorescence channel (green in merged/composite frames). Merged—200X: merged form of the phalloidin and Hoechst epi-fluorescence images obtained at two-hundred-fold magnification; merged with PH—200X: merged form of two-hundred-fold magnification epi-fluorescence images with the corresponding phase-contrast (PH) image for the region under micro-piston; merged with PH—100X: merged form of one-hundred-fold magnification epi-fluorescence images with the corresponding phase-contrast (PH) image for the region under and around micro-piston in channel. (TIF) [file pone.0279896.s004.tif]

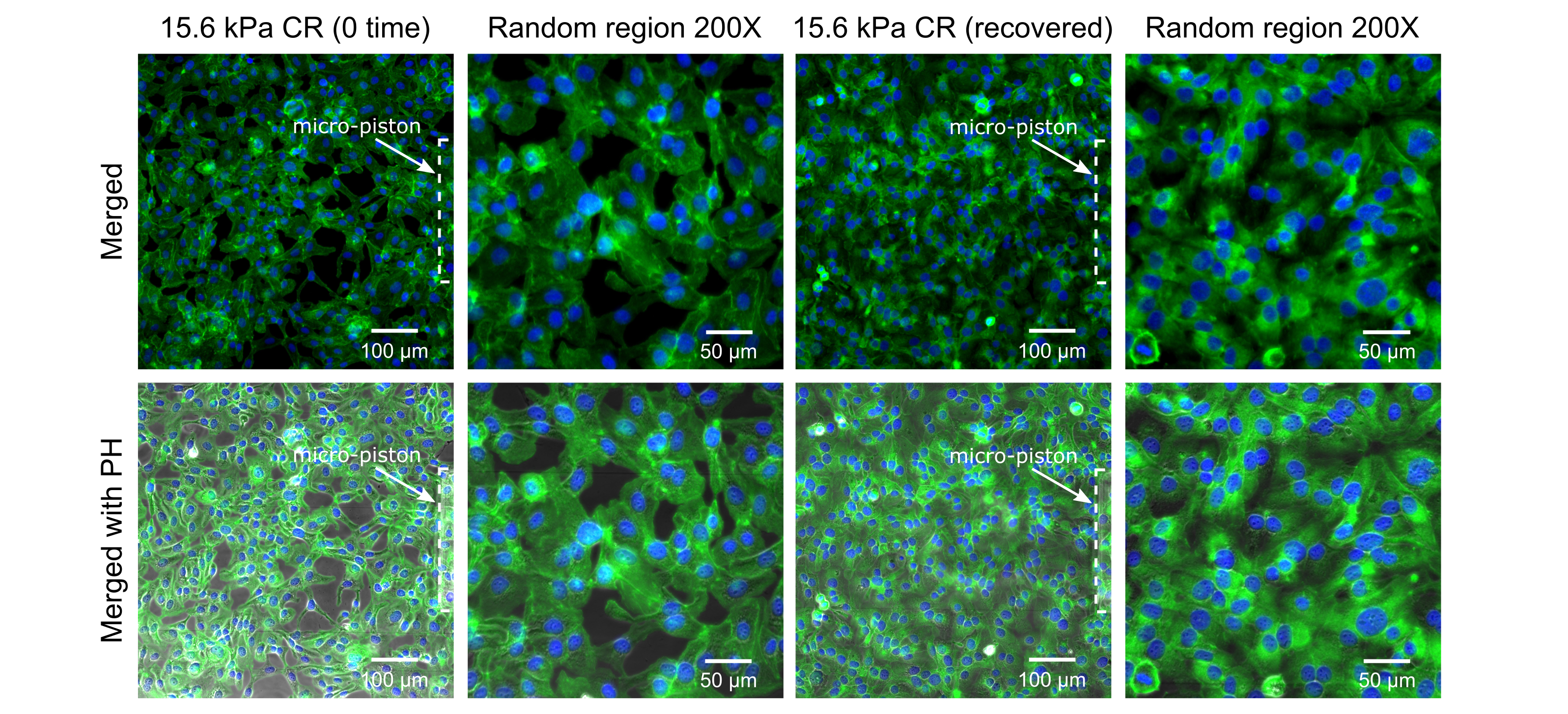

Supplement: S5 Fig — Control cell groups stained for actin (green) and nuclei (blue) for their form at zero time and 24 h-recovery. Merged: merged form of the phalloidin (stain for actin) and Hoechst (stain for nuclei) epi-fluorescence images; merged with PH: merged form of the epi-fluorescence images with the corresponding phase-contrast (PH) image; Random region 200X: two-hundred-fold magnification images of control cells in random region as part of the control region. (TIF) [file pone.0279896.s005.tif]

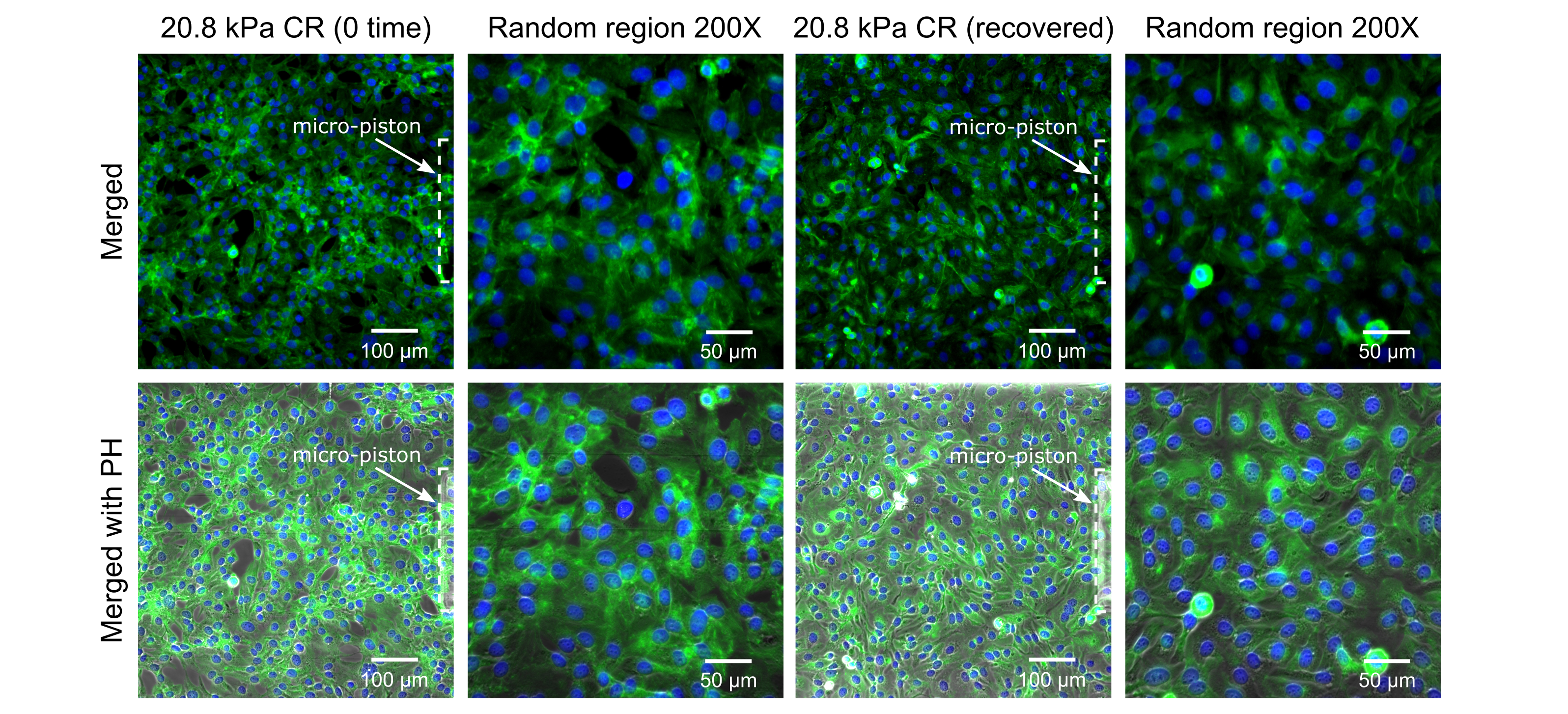

Supplement: S6 Fig — Control cell groups stained for actin (green) and nuclei (blue) for their form at zero time and 24 h-recovery. Merged: merged form of the phalloidin (stain for actin) and Hoechst (stain for nuclei) epi-fluorescence images; merged with PH: merged form of the epi-fluorescence images with the corresponding phase-contrast (PH) image; Random region 200X: two-hundred-fold magnification images of control cells in random region as part of the control region. (TIF) [file pone.0279896.s006.tif]
