# Supplementary material for: Application of sequential cyclic compression on cancer cells in a flexible microdevice
Source: PLoS One. 2023 Jan 5;18(1):e0279896. doi: 10.1371/journal.pone.0279896 (PMC9815655; doi:10.1371/journal.pone.0279896)
Supplement: Attachment — Submitted filename: Response to Reviewers.pdf [file pone.0279896.s012.pdf]

College of Engineering

UNIVERSITY OF CANTERBURY
Te Whare Wānanga o Waitaha
CHRISTCHURCH NEW ZEALAND

Department of Electrical and Computer Engineering
University of Canterbury
Private Bag 4800, Christchurch
NEW ZEALAND

Tel: +64 3 364 2987, Fax: + 64 364 2761

July 7, 2022

The Editor
PLOS One
PLOS Publishing

DETAILED RESPONSES TO THE JOURNAL AND REVIEWERS

Reviewers' questions are underlined.
Authors' responses are given in bold font.
Blue highlighted: Previous or existing parts in the previously submitted manuscript.
Green highlighted: Revised or newly added parts in the revised version of the manuscript.

Journal Requirements:

When submitting your revision, we need you to address these additional requirements.

1. Please ensure that your manuscript meets PLOS ONE's style requirements, including those for file naming. The PLOS ONE style templates can be found at https://journals.plos.org/plosone/s/file?id=wjVg/PLOSOne_formatting_sample_main_body.pdf and https://journals.plos.org/plosone/s/file?id=ba62/PLOSOne_formatting_sample_title_authors_affiliations.pdf

We improved the order of the sections, located Supporting information at the end of the manuscript (after References) as shown in https://journals.plos.org/plosone/s/file?id=wjVg/PLOSOne_formatting_sample_main_body.pdf

We improved the format of equation (1) in section "Imaging and data analysis".

We added initials at the end of corresponding authors' emails.

We checked figure citations in text and file naming.

We improved listing of our references according to PLOS ONE guidelines.

2. Thank you for stating the following in the Acknowledgments Section of your manuscript:
[The authors thank Helen Devereux, Gary Turner, and Nicole Lauren-Manuera for assistance. We also thank Mathieu Sellier for help with COMSOL, and Campbell Sheen for assistance with the EPA protocol to use the Actin-GFP BacMam construct. Financial support was provided by the MacDiarmid Institute for Advanced Materials and Nanotechnology and the Biomolecular Interaction Centre. Additional funding for V.N. was provided by Rutherford Discovery Fellowship RDF-19-UOC-019. We also thank University of Canterbury for Faculty of Engineering PhD Publishing Scholarship to S.O.]

We note that you have provided funding information that is not currently declared in your Funding Statement. However, funding information should not appear in the Acknowledgments section or other areas of your manuscript. We will only publish funding information present in the Funding Statement section of the online submission form. Please remove any funding-related text from the manuscript and let us know how you would like to update your Funding Statement. Currently, your Funding Statement reads as follows:
[Financial support was provided by the MacDiarmid Institute for Advanced Materials and Nanotechnology and the Biomolecular Interaction Centre. Additional funding for V.N. was provided by Rutherford Discovery Fellowship RDF-19-UOC-019. S.O. also thank University of Canterbury for Faculty of Engineering PhD Publishing Scholarship.]

Please include your amended statements within your cover letter; we will change the online submission form on your behalf.

Acknowledgments section has been revised in the manuscript as follows:

“The authors thank Helen Devereux, Gary Turner, and Nicole Lauren-Manuera for assistance. We also thank Mathieu Sellier for help with COMSOL, and Campbell Sheen for assistance with the EPA protocol to use the Actin-GFP BacMam construct.”

Update for our Funding Statement has been requested in the cover letter as in the following:

[Financial support was provided by the MacDiarmid Institute for Advanced Materials and Nanotechnology and the Biomolecular Interaction Centre. Additional funding for V.N. was provided by Rutherford Discovery Fellowship RDF-19-UOC-019. We also thank University of Canterbury for the Faculty of Engineering PhD Publishing Scholarship to S.O.]

[Note: HTML markup is below. Please do not edit.]

Reviewers' comments:

Reviewer's Responses to Questions

Comments to the Author

1. Is the manuscript technically sound, and do the data support the conclusions?

The manuscript must describe a technically sound piece of scientific research with data that supports the conclusions. Experiments must have been conducted rigorously, with appropriate controls, replication, and sample sizes. The conclusions must be drawn appropriately based on the data presented.

Reviewer #1: No

Reviewer #2: No

Reviewer #3: Yes

2. Has the statistical analysis been performed appropriately and rigorously?

Reviewer #1: I Don't Know

Reviewer #2: No

Reviewer #3: Yes

3. Have the authors made all data underlying the findings in their manuscript fully available?

The PLOS Data policy requires authors to make all data underlying the findings described in their manuscript fully available without restriction, with rare exception (please refer to the Data Availability Statement in the manuscript PDF file). The data should be provided as part of the manuscript or its supporting information, or deposited to a public repository. For example, in addition to summary statistics, the data points behind means, medians and variance measures should be available. If there are restrictions on publicly sharing data—e.g. participant privacy or use of data from a third party—those must be specified.

Reviewer #1: Yes

Reviewer #2: Yes

Reviewer #3: Yes

4. Is the manuscript presented in an intelligible fashion and written in standard English?

PLOS ONE does not copyedit accepted manuscripts, so the language in submitted articles must be clear, correct, and unambiguous. Any typographical or grammatical errors should be corrected at revision, so please note any specific errors here.

Reviewer #1: Yes

Reviewer #2: Yes

Reviewer #3: Yes

5. Review Comments to the Author

Please use the space provided to explain your answers to the questions above. You may also include additional comments for the author, including concerns about dual publication, research ethics, or publication ethics. (Please upload your review as an attachment if it exceeds 20,000 characters)

Reviewer #1: Cancer mechanics are important for tumor biology and progression, but the effects of mechanical forces on cancer cells are poorly understood. The authors present methodology for applying compressive forces to cancer cells using a microfabricated platform. They show that cyclic compression affects the intensity of actin-GFP and nuclear morphology in ovarian cancer cells. Although this is an interesting system, there are a number of issues related to novelty, biological relevance and interpretation that need to be addressed.

Specific comments:
1) In the introduction, it is mentioned that "...cells are exposed to chronic compressive stress from different sources such as tumour growth, displacement within stromal tissue and hydrostatic pressure out of ascitic fluid in peritoneal cavity." This is not completely accurate, as hydrostatic pressure will not deform the cell in the same way as anisotropic compression. The forces would be isotropic for fluid pressure.

Asem *et al.* (2020) Scientific Reports, show that ascites-induced compression exist in the peritoneal microenvironment due to the ascites volume that can reach >2L and cause intraperitoneal pressure (IPP) to rise from normal values of 5 mmHg to as high as 22 mmHg (~3 kPa). Ovarian cancer cell adhesion to peritoneum increased due to the impact of ascites-induced compression, shown in *in vivo* assay.

Asem *et al.* also applied a pressure of ~3 kPa on OVCAR5 or OVCAR8 cells added atop the mesothelial surface of murine peritoneal explants ex vivo for 24 hours in a bulk compression system. As a result, they observed enhanced interaction between peritoneal mesothelial cells and cancer cells via induction of tunneling nanotubes (TNT), which later lead to metastatic ovarian cancer progression. These compression-induced TNTs are actin based and aid in trafficking of mitochondria between ovarian cancer cells and mesothelial cells.

The other consequence of the ascites-induced compression through hydrostatic pressure in the peritoneal cavity has been a more linear anisotropic alignment of peritoneal collagen fibers. Such alignment of collagen is a common indication for enhanced invasion of solid tumors.

Overall, Asem *et al.* have found that ascites-induced compression promotes metastatic ovarian cancer progression.

This information has been summarized and added in the first paragraph of the Introduction: "…….Ascites-induced compression exists in the peritoneal microenvironment due to the ascites volume that can reach >2L and increases ovarian cancer cell adhesion to peritoneum, shown by Asem *et al.* using *in vivo* assay [7]. This peritoneal microenvironment is continuously affected by the movements of the surrounding organs, musculoskeletal dynamics, and gravity [6]……. "

2) The rationale for using matrigel is not clear. Collagen and/or fibronectin would be more representative.

In the Materials and methods section "Cell culture, Matrigel coating and chip loading with cells" it was noted that "…… Matrigel was previously shown to facilitate cancer cell adhesion and mimic the extracellular matrix (ECM) microenvironment in comparison to other substrates ….. ."

It has been also added into this section and further referenced that "Matrigel is commonly used basement membrane extracellular matrix for providing improved microenvironmental and physiological conditions in *in vitro* culture assays of cancer cells by its composition [20-23].", as shown by such as Kleinman et al (1982) Biochemistry, Mullen et al (1996) Int J Cancer, Kleinman et al (2005) Seminars in Cancer Biology, and Vaillant et al (2011) Breast Cancer.

Major components of Matrigel are laminin, collagen IV, heparan sulphate proteoglycans, entactin and nidogen (Kleinman et al.,1982, Biochemistry). It also contains various growth factors such as TGF-β, epidermal growth factor, fibroblast growth factor and tissue plasminogen activator, insulin like growth factor which are naturally occurring in tumor microenvironment (Corning Inc.). Thus, Matrigel is rich in extracellular matrix proteins and polymerises under normal physiological conditions to produce a reconstituted, biologically active matrix material which is effective for the attachment and differentiation of cellular material as shown by Kleinman et al (1982) Biochemistry.

It has been also added in Conclusions "Similar to Matrigel coating used in this study, our platform and established chip loading method would allow coatings of other hydrogel types such as collagen and fibronectin", which could be tried as in the method shown in Figure 1(b).

3) Does the disruption of the cytoskeleton depend on the rate of application of the force? If the piston is applied more slowly, do the cells have time to adjust and avoid damage?

Regarding deformation and disruption of actin cytoskeleton, Figure 3 and Figure 4 show how live cell actin cytoskeleton behave under dynamic compression applied over time in cyclic mode at a physiological value and sequentially in varying applied pressures in ascending order, and are out of at least 3 independent experiments. In text, while explaining the associated figures, the representative pressure profile for sequential cyclic compression process was indicated to be in Figure 1(d): "Similar as to the sequence shown in Fig 1(d), first, a pressure amount ladder was formed up to a pressure in the mild compression range, represented by an externally applied pressure of 350 mbar and resulting in a piston contact pressure of 15.6 kPa, during which the piston reached the cells at the bottom surface and cells started to distinctly deform."

We think that this pressure application is gradual enough for cells to adjust to the varying pressures during sequential cyclic compression application. Mechanical cell damage that we see at higher pressures is intrinsic to cell response to higher amounts of applied pressure. The following explanation has been added at the end of the 1st paragraph in section "Live cell actin profiles of cancer cells during sequential cyclic compression":

"Throughout the application of sequential cyclic compression, cells were allowed to adjust to each next higher applied pressure. As shown in the pressure amount ladder in Fig 1(d), pressure was applied gradually in 30 s among rising steps of the ladder until the piston reached the focal plane of the cells and started cell deformation. After observing that cells were distinctly deformed at a mild compression level but not to a disruption or lysis level, compression was set to be in cyclic mode. Although the pressure was changing suddenly along the cycles as per the settings of the selected cyclic mode in the pressure controller, cells were not damaged when compressed as they resulted in being resistant at this physiological value. Next, pressure application was continued with gradual increase from 350 to 370 mbar, from 370 to 400 mbar and from 400 to 640 mbar in 30 s for each, as shown in Fig 1(d). Thus, cells could have the time to adjust during the pressure increase and show their intrinsic response to the pressurized stage of each compression level."

4) In Figure 3, it looks like some labeled cells are actively moving away from the area of the piston. Did this affect the measurements? Related to this, the authors might refer to doi.org/10.1073/pnas.1118910109.

For CTCF calculations as shown in Eq (1) (format of the equation has been revised) in methods section "Imaging and data analysis", while analysing the cells under micro-piston we must draw an ROI and set it as threshold that separates the analysis area (Area of micro-piston ROI) from the rest which is control group. We set an ROI from the beginning and then we do not change its boundaries during the frames taken along the stages of the dynamic compression process. The way we decided the micro-piston ROI is shown as in the ROI (yellow frame) drawn the videos in the links, we accept slightly larger area which is drawn based on the pressurized from of the micro-piston.

Phase contrast with micro-piston ROI:
https://www.dropbox.com/s/sixcpmtw2abteth/S300um_3pistonSKOV-3%20actin-GFP_phasecontrast_withROI.avi?dl=0

Actin-GFP with micro-piston ROI:
https://www.dropbox.com/s/1ww7p90el7t3ej1/S300um_3pistonSKOV-3%20actin-GFP_withROI.avi?dl=0

Cells are partially compressed partially not under the micro-piston periphery (boundaries of the ROI), so in CTCF calculations the compressed and then buckled

back parts of the cells were taken into account. This was explained as in the last paragraph in section "Live cell actin profiles of cancer cells during sequential cyclic compression": "The current device also allowed the response of individual cells at the interface between compressed and non-compressed regions in the periphery of the micro-piston to be studied, thus providing a degree of control of the localization of compression (see S2 Movie). As depicted in the video, parts of cells at the interface were subject to compression, while the rest remained uncompressed. Thus, the cell actin was deformed and damaged only partially in the compressed part depending on the magnitude of the applied pressure, whereas it remained more intact in the non-compressed part. This functionality which is unique to our platform by having control and compressed groups in the same microdevice, is useful to mimic partial cellular deformation and damage that can occur *in vivo* in presence of a localized mechanical loading."

Cells under the periphery of the micro-piston are mainly changing shape in partial and some are being pushed away at compressed stage due to the physical presence of the micro-piston edges and accumulated cell debris towards the end of process when higher pressures were applied. For the cells that might be moving away from the area of the micro-piston, such as the cell at the top right corner of the representative actin-GFP video in S2 Movie (in the links here as well), which looks pulled its body part and changed its shape to move away from the contacting piston, the reviewer is right, such cells might get impacted by the pressurized pressure and sensing the mechanical stress loading and move away from the stress. However, to better understand this sensing and movement of the cells away from the stress (whether this is a mechanism for the cell to protect itself from the mechanical stress) we need to observe more cells doing that. On the other hand, we are more confident on that majority of the cells were deforming, being pushed when the piston was contacting and then buckling back when the stress was removed and remained there as partially compressed and damaged (at high pressures towards the end of the process) and partially intact at the control region (that was outside the micro-piston ROI as threshold area).

Regarding movement of the cells away from under the micro-piston periphery, we suspect that confluency would be important as well. We expect that if the culture is confluent enough cells might not find enough space to move away, while in the less confluent cultures cells would be able to show their response better to decide whether to stay partially under the pressure or move away to a potentially available empty space nearby. We see the reviewer's observation invaluable to be further investigated in such confluency-controlled samples under compression with tracking of the cells at the periphery of the micro-piston in the future work.

5) Are the differences in cell response (and death) seen in a given compression experiment related to cell cycle?

We have not looked at the impact of compression on cell cycle in our experiments in this manuscript. However, we have extensively studied impact of compression on cell viability, running multiple assays in our previous reports (e.g. Onal et al

2021 Frontiers in Physics). Pressures that cause cell death and thus decrease in cell viability were well characterized there and thus these were referenced here at the beginning of the section "Live cell actin profiles of cancer cells during sequential cyclic compression": "For this, the actin-GFP transduced cancer cells were dynamically stimulated and compressed with various intermediate actuation pressures in a sequential cyclic manner, namely following a sequence from Mild (15.6–15.9 kPa) to Intermediate 1 (23.8–26.8 kPa), Intermediate 2 (37.8–41.7 kPa) and Severe (127.8–140 kPa) piston contact pressures. The first two ranges of this sequence fell within physiological and upper physiological pressure values and cell viability was high (on average 94 % for Mild and 77 % for Intermediate 1), while Intermediate 2 and Severe levels constitute hyperphysiological values and cell viability decreased (on average 43 % for Intermediate 2 and 20 % for Severe) [5]."

Thus, our previous report showed mechanical stress induced cell damage and death towards higher pressures so categorized as Intermediate 2 and Severe pressures, providing the degree of the damage in detail in Onal et al 2021 Frontiers in Physics. Cell viability results for the applied pressure ranges were reminded from Onal et al, 2021, Frontiers in Physics to give an idea at what viability states the cells were at these pressure ranges while investigating live cell actin cytoskeleton under dynamic compression in the current manuscript.

For the cell cycle itself e.g. impact of mechanical compression on mitosis, or other cell cycle phases, this looks beyond the scope of current work, yet, worth looking at in future work.

6) In Fig 6 b, it appears that there is a difference between the two "0 (CR)" values. Was this significant, and can the authors discuss the details of these samples?

All the statistical significance values for nuclear deformations in compressed and recovered cells after compression are noted in S2_File, newly added as supplementary file.

S2 File caption "Statistical significance levels among all groups, obtained with Student's t-test run for nuclear deformations in compressed and recovered cells after compression presented in Fig 5 and Fig 6." has been added. Fig 5 and Fig 6 captions have been also updated.

We added the following result and discussion for the difference between the two "0 (CR)" values: "There is statistically significant change in nuclei area between the control groups of zero-time and of 24 h-recovery after compression (see S2 File). Although these are not exactly the same control groups in the same conditions and we intend to compare the test group (e.g. zero-time or 24 h-recovery after compression) with their own control groups, we consider that the observed significant difference could be due to cell-cell signaling between the cells in compressed and control groups during that 24 h-recovery period after compression. In the groups of zero-time after compression, we did not allow

control and compressed cells enough time to further interact with each other as they were fixed right after the compression."

We further added the following discussion for the absence of statistical change in nucleus area between the control and compressed groups at zero-time after compression, in the associated paragraph: "While we expect a statistically significant change between the compressed and its control group at zero-time after compression at 20.8 kPa similar to the results observed in our previous report [5], we attribute the absence of such a significant change to cells being cultured on Matrigel-coating here as opposed to PLL-coating used in the previous experiments. Since Matrigel is expected to better represent the cancer cell microenvironment, it thus aided in cell growth into denser monolayers and formation of supporting extracellular matrix layer, that can alter sensing of the applied stress for the compressed cells."

7) The meaning of "plasticity" should be defined more clearly. Does plasticity refer to mechanical lack of elasticity? If so, why would we expect this to be related to loss of actin-GFP? Also, plasticity would be reflected by a lack of recovery of the cell to its original state. Or are the authors simply analyzing biological processes that restore the cell after stress?

For analysis of actin-GFP signal we had done CTCF calculations and have now improved the graph to show the statical significances in Fig 4 in the revised manuscript. When there was statistically significant loss in actin-GFP signal and as seen on S2 Movie, actin disruption happens and that indicates for plastic response of the cells at those higher pressures. We added a discussion in the last paragraph of section "Live cell actin profiles of cancer cells during sequential cyclic compression": "The observed disruption of actin cytoskeleton in these experiments indicates for cell plasticity during a cyclic compression in ovarian cancer when varying higher pressures occur in the microenvironment. There is need for more studies in literature to compare our results on cell plasticity after cyclic or dynamic compression applied sequentially up to high pressures. For cell actin deformation at milder pressures, however, a distinct change in actin cytoskeleton morphology was observed by Asem *et al.* for mesothelial cells, interacting with ovarian cancer cells, under compression that is statically applied at ~3 kPa for 24 h in a bulk compression system. As a result, the interaction between ovarian cancer cells and mesothelial cells by enhanced formation of actin-based tunneling nanotubes (TNTs), and hence metastatic progression in ovarian cancer, was promoted under compression [7]."

In the section Conclusions it has been also added that "While actin deformation at mild pressures (e.g. 15.6 kPa) in cyclic mode indicates for cell elasticity, the resultant actin disruption indicates for cell plasticity that can emerge when ovarian cancer cells are exposed to varying and higher pressures in a microenvironment."

The reviewer is right on that plasticity refers to that mechanical lack of elasticity. If there is a lack of recovery of the cell to its original state due to, for instance, actin disruption, we think this is because of cell plasticity (plastic response of cells), as well. However, the cell being not recovering to its original state does not always refer to cell plasticity (mechanical lack of elasticity), especially at lower intermediate pressure values such as 20.8 kPa. The cell could be adapting to this pressure level and acquire a new phenotype while it is still elastic despite that there is an incomplete shape recovery to its original state.

We have gone through the entire manuscript and checked the parts where elasticity, plasticity and recovery words are used and revised the associated parts accordingly to prevent confusions in case of that the terms had been used interchangeably.

Thus, wherever further slight changes were done, those are also highlighted in the revised sentences as in the following.

The following sentence in the last paragraph of Results and discussion has been revised:
"Furthermore, the data obtained in this work suggest that ovarian and likely other cancer cells can exhibit a plastic cell response to applied sequential cyclic compression."

revised to

"Furthermore, the data obtained in this work suggest that ovarian and likely other cancer cells can exhibit a varying cell response to applied sequential cyclic compression."

In the paragraph starting as in the following in Introduction:
"In the presence of a mechanical stress, cells strive to restore their shape to maintain cell integrity after the removal of mechanical loading. However, cells may show a plastic response in the form of cytoskeletal bond ruptures and an incomplete cell shape recovery [1]. Meanwhile, cells produce an adaptive mechanism via these bond ruptures to reduce the mechanical cell stress and thus protect themselves against mechanical damage, while subsequently deforming under the mechanical load [1,8]."

3rd sentence has been revised to

"Meanwhile, cells produce an adaptive mechanism to reduce the mechanical cell stress and thus protect themselves against mechanical damage, while subsequently deforming under the mechanical load [1,10]."

Plastic word has been removed in the following sentence in Introduction:

“Using a single cell microfluidic compression platform, their application resulted in full recovery and thus no permanent ~~plastic~~ deformation in MCF10A normal breast epithelial cells after compression [2].”

Plastic word has been removed in the following sentence in Results and discussion section “Actin cytoskeleton and nuclei profiles of cancer cells after compression and recovery”:
“No statistical difference in cell height before and after compression was observed, indicating that the breast epithelial cells did not exhibit permanent ~~plastic~~ deformation.”

8) Similarly, the meaning "recovery" should be defined more clearly. The authors write "The extent of recovery of compressed cells, inferred by imaging cell membrane bulges and actin cytoskeleton and measuring the shape descriptors of cell nuclei, can give insights into the plasticity of cancer cells." However, there are no measurements of cell membrane bulges, and no spatial analyses of actin organization. How do we know the cells have "recovered"?

Our response to this question is in agreement with the response to previous (7th) question of the reviewer.

Indeed, we used the recovery word in this manuscript for the investigation of cell response after compression, whether they fully restore their shape or not, allowing cells enough time. Thus, it refers to a process to investigate what happens to cells after being exposed to compression.

To prevent confusion the sentence in the last paragraph of Introduction has been revised to a clear form: “The extent of recovery of compressed cells can give insights into the cell integrity and adaptation of cancer cells to restore their shape or acquire new one when exposed to mild or upper mild compressive stress at physiological levels.”

Cell membrane bulges formed at compressed stages are distinct in S1 Movie which include huge amount of information per time-lapse imaging video. While membrane bulges in cells observed in phase contrast live cell imaging are fixed directly after compression and distinctly appeared as increased actin signal at cell edges when fluorescently stained, their further analysis out of time-lapse videos would require an advanced and automated image analysis method as label-free and high-content. Spatial analyses of actin organization (in addition to CTCF calculations) would require imaging data taken at higher resolution, via a super resolution microscopy for instance.

9) Other groups have published similar pressure-actuated devices for compressing cells, so there should be more discussion of these studies and comparison with the presented

technology. In general, a more extensive literature review on in vitro analyses of cancer cell solid stress would be useful.

The following paragraph that was in Results and discussion has been moved to Introduction and slightly revised:

"To date, dynamic compression experiments in literature include frequencies of 0.1 – 30 Hz with an applied stress of 5.1, 9.3, 12.9 and 18.7 kPa on breast cancer cells over short durations (30 – 300 s), as used by Takao *et al.*, who observed a mixed mode of apoptosis and necrosis-dominant cell death in mechanically compressed monolayers in a bulk compression platform [18]. In contrast, work by Novak *et al.* used a frequency of 0.05 Hz for cyclic compression applied at 3.9 – 6.5 kPa for 24 hours [3]. They observed an increase in proliferation capacity and decrease in apoptosis for OVCAR3 and OVSAHO ovarian cancer cells cultured in 3D hydrogel components in a bioreactor at millimeter scales. In another example, Ho *et al.* applied cyclic compression by alternating between external pressures of 10 and 15 psi (68.9 and 103.4 kPa) at 0.5 Hz for 6 minutes [2]. Using a single cell microfluidic compression platform, their application resulted in full recovery and thus no permanent plastic deformation in MCF10A normal breast epithelial cells after compression [2]. In our work we add to these examples by using the capabilities of our compression platform to apply cyclic compression with a low frequency in microfluidic settings. In particular, we utilize this functionality to provide a first investigation of the effects of cell compression, deformation and recovery after compression on SKOV-3 ovarian cancer cells. As part of this we provide evidence that the amount of the pressure and duration of the application clearly affects morphology during cell compression and recovery. While compression frequencies in an *in vivo* tumor microenvironment are not yet fully known, cyclic compression is important for maintenance of cell culture during mechanical compressions in an *in vitro* experimental setup, in particular as it enhances media flow underneath the micro-pistons."

10) Given the relatively rapid application and removal of compressive forces, there must be some interesting fluid dynamics happening between the cells and the piston. Can the authors estimate the changes in fluid pressure and shear stress on the cells? This could also affect the cell biology.

This is very good point. We have not particularly looked at the shear stress effect. We do not have horizontal, continuous flow on the cells in the channel, which means the cultures were in static media, but while moving the piston at vertical direction, the media also displace and that might create a negligible shear stress. However, we believe that the solid stress that we applied with physically sharply contacting surface of the micro-piston dominates the effect of compressive stress on cells, not that of shear stress. Considering other stresses from the very beginning, we applied the pressure gradually on the cells as demonstrated at the beginning of S1 Movie while increasing the pressure at a gradual rate shown on the

pressure ladder on sensor reading graphs. Cells were experiencing the changing pressure mostly at vertical directions in the form of compression.

There could be hydrostatic pressure which also translate into compressive stress while deflecting the membrane, so we expect that hydrostatic pressure to be all around the channel. Comparing the viability results among the different shapes and among different compression states, studied extensively in Onal et al. 2021 Frontiers in Physics, the cells in the control regions around the micro-piston were intact and alive throughout the applications as well as that the cells under micro-piston responded according to the applied pressure. This shows that other stress factors seem to not have an influence on cells in terms of viability.

If we had a horizontal cell compression setup as Paggi et al. (2020, Sensors Actuators B: Chemical) had in their design with a vertical membrane adjacent to the cell culture chamber that is deflected at horizontal direction, we would need to be looking at multi-modality of the forces and the combination of compressive and shear stress as they did in their work. However, in our design, the membrane is located horizontal to the cell culture chamber, and we have a piston to physically contact the cells once compressed. Thus, we deflect the membrane vertically and the forces are mainly in compressing mode at the vertical direction in the cell culture chamber.

Nevertheless, we believe that the reviewer made an interesting point here, worth looking at combination of shear stress and compressive stress effects on cells further in the scope of a next piece of work within cell compression field.

11) Further justification should be provided for the cyclic stress schedule chosen. Musculo-skeletal tissues would be expected to experience such forces, but this is not clear for ovarian cancer.

The cyclic stress schedule (i.e. 5-min compressed stage followed by 5-min rest stage in each cycle) was chosen to allow cells enough time during each stage to complete bulge formation or recovery, processes which were considered important for the investigation of cell deformation and recovery after compression. Membrane bulge formation and recovery processes are clearly observable in S1 Movie. This was explained in the following paragraph in section "Comparison of the experimental and computational results in micro-piston device"

"While inferring these results, the cell compression process was imaged in detail in Matrigel-coated microchannels at a speed of 1 frame per 100 ms, as shown in S1 Movie. As illustrated by this time-lapse video, the platform allowed for the temporal evolution of the dynamic cell compression to be investigated in each cycle while the piston was contacting cells and applying compression. Distinct cell membrane bulges could be observed to form during such compression at piston contact pressure of 15.6 kPa and recovery of cell membranes from these bulges was visible when pistons were lifted off during rest stages. Although the platform is capable of

temporal control of cell compression (e.g. to decrease, increase or remove the pressure at any certain time interval), duration of each stage in a cycle was set to 5 minutes, corresponding to a 5-min compressed stage followed by 5-min rest stage in each cycle. This was chosen to allow cells enough time during each stage to complete bulge formation or recovery, processes which were considered important for the investigation of cell deformation and recovery after compression. Sensor readings in S1 Movie showed that the micro-piston actuated according to externally applied pressures with reliable control and could be operated in short durations gradually or suddenly when needed."

Further explanation and discussion have been also added in this section as in the following paragraph:

"Cyclic compression in the study of Ho *et al.* was applied only for 6 minutes in total [2], significantly shorter than the entire duration of a cyclic compression in this work, and results presented here suggest that cell deformation at compression stage and recovery at rest stage depends on the temporal length of the compression on cells. Particularly observable in S1 Movie, cell membrane bulge formation slowly developed during the compression stage while cells were responding to contact pressure at the interface with the PDMS piston. This was despite the fact that the piston was actuated onto the cells rapidly during cyclic compression as directed by the external pressure controller. As shown by the pressure sensor readings in the same movie, PDMS membrane and piston were consistently responding to amount, duration and mode of the applied pressure. Thus, the gradual deformation of cells and bulge formation during compression stages must originate in the response type of the cells to mechanical load when in solid contact with the PDMS piston. During rest stages, where the compression was lifted, cell bulges did not fully recover right after compression was removed, suggesting that the cells require a certain amount of time to recover compressed cellular parts (S1 Movie). Based on these observations of temporal evolution of the cell compression application, and comparison of application durations in this study with the study by Ho *et al.* [2], we hypothesize that this recovery also depends on the duration and number of the cycles."

Regarding sources of compression ovarian cancer cells are exposed to, further information has been added in Introduction:

"While some cells experience permanent plastic deformation after a repetitive mechanical tensile loading and unloading, the impact of such repetitive compression on deformation of cells remains largely unknown [1,2]. As such, the ability to apply cyclic compression is crucial for any experimental setup aimed at the study of mechanical compression taking place in cell and tissue microenvironments [3–5]. For instance, in ovarian cancer, cells are exposed to chronic compressive stress from different sources such as tumor growth, displacement within stromal tissue and hydrostatic pressure out of ascitic fluid in peritoneal cavity [3,6]. Ascites-induced compression exists in the peritoneal microenvironment due to the ascites volume that can reach >2L and increases

ovarian cancer cell adhesion to peritoneum, shown by Asem *et al.* using *in vivo* assay [7]. This peritoneal microenvironment is continuously affected by the movements of the surrounding organs, musculoskeletal dynamics, and gravity [6]. Thus, ovarian cancer cells might experience cyclic compression profiles during chronic exposure to compression from different sources and due to anatomical location of the ovary. Mimicking such compression profiles and physiological pressure values in an *in vitro* dynamic compression system is necessary to further study impact of compressive stress in cancer.

Compressive stress is estimated to reach 18.9 kPa for human tumors and can exceed 20 kPa based on the experimental data from murine tumors, while those values can be even higher in situ with the impact of the surrounding extracellular matrix in tumor microenvironment [3,8]. Compared to other cancer types such as breast and metastatic bone niches, compression studies have been limited for ovarian cancer, including for investigating the impact of various amounts of applied pressures [6]. Pressure values existing in literature that are applied for ovarian cancer have been in a range from ~3 kPa to 6.5 kPa [3,7,9]. Ovarian cancer cell response at higher applied pressures that can occur in the body need further investigation."

Furthermore, it was noted in our manuscript that "While compression frequencies in an *in vivo* tumor microenvironment are not yet fully known, cyclic compression is important for maintenance of cell culture during mechanical compressions in an *in vitro* experimental setup, in particular as it enhances media flow underneath the micro-pistons."

Reviewer #2: The following are the attached Comments for Authors.

In this manuscript, authors characterized changes and recovery in actin fluorescence signal and nucleus shape after applying cyclic compression to ovarian cancer cells. However, there are several major issues with the current manuscript: 1) the introduction lacks information and citations of previous work and does not address the importance of this work, i.e., why apply cyclic compression on ovarian cancer cells; 2) Key parts of the experimental design lack rationale, i.e. why 5 min pressure 5 min recovery, how large is the piston/cell contact area, variability of the pressure within contact area, etc. 3) Several results are questionable, as detailed the following comments; and 4) Several results lack information on significance tests and number of independent studies. As such, the reviewer recommends rejection.

1. The introduction is not clear what the similarities and differences are between chronic and cyclic compression, and why cyclic compression is important to study in ovarian and other cancer cells.

Chronic compression in this manuscript and in literature was meant to be that ovarian cancer cells are under a dynamic compression from different sources.

Chronic word was removed from the following sentence to prevent confusion, as it was meant to be that higher pressure amounts (total perceived stress which can originate from chronic exposure to compression from different sources) and profiles that can be experienced by ovarian cancer cells need to be mimicked in an *in vitro* dynamic compression system and the impact on cells need to be studied further:
"Mimicking such ~~chronic~~ compression profiles and physiological pressure values in an *in vitro* dynamic compression system is necessary to further study impact of compressive stress in cancer."

The first two paragraphs of the Introduction have been revised as follows:

"While some cells experience permanent plastic deformation after a repetitive mechanical tensile loading and unloading, the impact of such repetitive compression on deformation of cells remains largely unknown [1,2]. As such, the ability to apply cyclic compression is crucial for any experimental setup aimed at the study of mechanical compression taking place in cell and tissue microenvironments [3–5]. For instance, in ovarian cancer, cells are exposed to chronic compressive stress from different sources such as tumor growth, displacement within stromal tissue and hydrostatic pressure out of ascitic fluid in peritoneal cavity [3,6]. Ascites-induced compression exists in the peritoneal microenvironment due to the ascites volume that can reach >2L and increases ovarian cancer cell adhesion to peritoneum, shown by Asem *et al.* using *in vivo* assay [7]. This peritoneal microenvironment is continuously affected by the movements of the surrounding organs, musculoskeletal dynamics, and gravity [6]. Thus, ovarian cancer cells might experience cyclic compression profiles during chronic exposure to compression from different sources and due to anatomical

location of the ovary. Mimicking such compression profiles and physiological pressure values in an *in vitro* dynamic compression system is necessary to further study impact of compressive stress in cancer.

Compressive stress is estimated to reach 18.9 kPa for human tumors and can exceed 20 kPa based on the experimental data from murine tumors, while those values can be even higher in situ with the impact of the surrounding extracellular matrix in tumor microenvironment [3,8]. Compared to other cancer types such as breast and metastatic bone niches, compression studies have been limited for ovarian cancer, including for investigating the impact of various amounts of applied pressures [6]. Pressure values existing in literature that are applied for ovarian cancer have been in a range from ~3 kPa to 6.5 kPa [3,7,9]. Ovarian cancer cell response at higher applied pressures that can occur in the body need further investigation."

The following sentence that was in Results and discussion has been moved to Introduction: "While compression frequencies in an *in vivo* tumor microenvironment are not yet fully known, cyclic compression is important for maintenance of cell culture during mechanical compressions in an *in vitro* experimental setup, in particular as it enhances media flow underneath the micro-pistons."

2. Page 2, line 9-11: "Such compression is estimated to reach 18.9 kPa for human tumours and can exceed 20 kPa based on the experimental data from murine tumours [3, 7]." Given the wide range of compressive stresses in different tumor models & species, the authors should specify the physiological range of compression for ovarian cancer.

Regarding ovarian cancer, compression sources and current studies in literature and expected compression values are now explained in the first two paragraphs of the Introduction in our revised manuscript. Further compression values and findings for ovarian cancer cell compression based on the ovarian cancer cell response to the applied pressures in our study are presented in Results and discussion in this manuscript.

3. The introduction is not clear about the similarities and differences between chronic and cyclic compression, and why cyclic compression is important to study in ovarian and other cancer cells.

Same as the 1st question.

4. The innovations in the microdevice authors developed are not clear. What has been developed in the past and what is new should be summarized in the Introduction.

Last two paragraphs of Introduction included information on what has been developed in past and what is new. Now the two paragraphs are revised, and the last paragraph has been further improved to better reflect on what is new.

"Thus, with the purpose of applying compression in a cyclic, dynamic and controlled manner for the investigation of cell deformation and recovery, we have recently developed a robust cyclic compression microfluidic method based on a flexible microdevice. This platform provides extensive control of the amount, duration, and mode within a pressure profile. Fabrication and characterization of the multilayer microfluidic platform, and the observation of directional growth of cells, viability and mechanical cell lysis as end point assays under static state and pressurized states within this platform were demonstrated previously [4,5]."

"In this paper, we further extend the reproducibility and repeatability validation of our flexible microdevice-based compression method via comparisons of experimental and computational piston actuations for independent microdevices with different membrane thicknesses. We then use the microfluidic platform for the *in vitro* application of cyclic cell compressions that mimic biologically relevant compression profiles occurring in cellular microenvironments. In particular, we demonstrate applicability of the platform for the chronic exposure of ovarian cancer cells to repetitive compressive stress. As such, the current work extends the use of the platform to the application of cyclic compressions at and beyond physiological pressure values in a sequential fashion to study dynamic biomechanical processes by recording GFP-tagged actin dynamics of live cells under compression. In relation to our previous work which discussed initial findings on cellular deformations observed at cyclically applied ascending pressures, the current work also expands applicability by providing a more physiologically relevant setting in form of a hydrogel coating within micro-piston device. We showcase data of cellular deformations and recovery during and after cyclic compression at mild (e.g. 15.6 kPa) and upper mild (e.g. 20.8 kPa) physiological pressure values, obtained via end point assays of actin and nuclei deformations in compressed cells at zero time or at 24 h-recovery after compression, showing flexibility and novel use of our microdevice for the applications of not only cell compression but also recovery. We demonstrate that the platform can control the strength and duration of cyclic compression, thus providing a powerful new tool for the study of mechanobiological processes, by which cell deformation, cytoskeletal and nuclear changes and recovery in cells exposed to such compression can be readily captured."

Revised to

"Thus, with the purpose of applying compression in a cyclic, dynamic and controlled manner for the investigation of cell deformation and recovery, we have recently developed a robust cyclic compression microfluidic method based on a flexible microdevice. Fabrication and characterization of the multilayer microfluidic platform, and the observation of directional growth of cells, viability and mechanical cell lysis as end point assays under static state and pressurized states

within this platform were demonstrated previously [4,5]. The developed platform was characterized to provide extensive control of the amount, duration, and mode within a pressure profile [5].

In this paper, we further extend the reproducibility and repeatability validation of our flexible microdevice-based compression method via comparisons of experimental and computational piston actuations for independent microdevices with different membrane thicknesses. We then extend the use of the platform to the application of cyclic compressions at and beyond physiological pressure values in a sequential fashion to study dynamic biomechanical processes by recording GFP-tagged actin dynamics of live cells under compression. To the best of our knowledge, this is the first demonstration of how live cell actin behave under dynamic compression applied over time in cyclic mode and sequentially at varying pressures in ascending order. Using time-lapse imaging, we thus show a sequence of actin deformation and disruption events based on the amount of the applied pressure in a flexible microdevice. The current work also expands applicability by providing a more physiologically relevant setting in form of a hydrogel coating within micro-piston device as shown in Fig 1. We showcase data of cellular deformations and recovery during and after cyclic compression at mild (e.g. 15.6 kPa) and upper mild (e.g. 20.8 kPa) physiological pressure values, obtained via end point assays of actin and nuclei deformations in compressed cells at zero time or at 24 h-recovery after compression. These experiments further prove flexibility and novel use of our microdevice for the applications of not only cell compression but also recovery. Differences observed between compressed cells fixed at zero time or after 24 h-recovery suggest that SKOV-3 cells exhibit deformations at the time of the compression, a proposed mechanism cells use to prevent mechanical damage. The extent of recovery of compressed cells can give insights into the cell integrity and adaptation of cancer cells to restore their shape or acquire new one when exposed to mild or upper mild compressive stress at physiological levels. As demonstrated with SKOV-3 ovarian cancer cells, biomechanical responses of cells to sequential cyclic compression and during recovery after compression can be revealed in a flexible microdevice where cell deformation, cytoskeletal and nuclear changes and recovery in cells exposed to such compression can be readily captured."

5. References supporting nucleus as mechanosensory are lacking. Kirby (2018) Nature Cell Biology, Fu (2012) Lab on a Chip, etc. Additionally, past findings on nucleus deformation in ovarian cancer cells and its functional effects should be introduced as background to this study

We thank the reviewer for letting us know about these references. We have looked at and cited them in the respective part of the Introduction "In itself, the nucleus is a mechanosensitive organelle [11-13] …..", along with Nava et al (2020) Cell.

We are not aware of ovarian cancer cell-specific cell nucleus deformation by mechanical compression and its functional affects if such study results exist in

literature. However, we have added a literature search from various cancer types, supporting background and motivation of this study:
"Morphological changes in nuclear structure, such as increased nuclear size, irregular shape by grooving, convolutions and invaginations of the nuclear envelope and altered organization by disturbed chromatin distribution, are commonly used cancer markers by pathologists [15]. Mechanical compression through confinement aid in mechanical adaptation of the cell as shown with Hela-Kyoto cancer cells in which stretching of the nuclear envelope and upregulation of actomyosin contractility were observed [16,17]. Furthermore, irregular nuclear morphology of cancer cells aligns with altered expression of nuclear envelope proteins such as lamins A/C or lamin B in cancers as detailed by Denais *et al.*, which has the capacity to promote metastatic processes [15]. Deformation of the large and stiff nucleus is required during the passage of metastatic cancer cells through the tight interstitial space and narrow capillaries in the microenvironment. Thus, mechanical properties of the cell nucleus, such as deformability and adaptation under compression, and its connection to the cytoskeleton may play a major role in cancer metastasis [15]."

6. Page 7, Fig 2(b): it is not clear which are experimental data and which are simulated pressures on the graph, and how well they match quantitatively.

Fig 2(b) was plotted for membrane thickness (on x-axis) vs. pressure sensor readings (on y-axis). Those values (both on x- and y-axis) are from experiments that resulted in a similar piston contact pressure. Before experiments those were computationally simulated as shown in Fig 2(a) to predict what external pressures need to be applied at different thicknesses to be able to get a similar piston contact pressure. Thus, while Fig 2(a) shows simulations, Fig 2(b) shows comparison of experimental values that were applied based on simulations. Because R^2 resulted to be high among independent experiments, giving a good correlation along different thicknesses and applied external pressures, to obtain a similar piston contact pressure calculated based on simulations, we think the model is robust to predict what external pressures should be applied when there is a variation in membrane thickness.

Caption of Fig 2(b) has been revised as in the following:
"(b) Correlation of independent experiments with the pressure sensor read for the externally applied pressure based on simulations run for the corresponding membrane thicknesses, resulting in a similar piston contact pressure."

Revised to

"(b) Correlation among independent experiments with the pressure sensor read for the externally applied pressures applied based on simulations in (a) run for the corresponding membrane thicknesses, resulting in a similar piston contact pressure."

The 2nd sentence in the following part at the end of the 1st paragraph in section “Comparison of the experimental and computational results in micro-piston device” has been revised to make it clear that the calculated R^2 was among independent experiments with different membrane thicknesses operated under different amounts of externally applied pressures that were predicted with simulations:
“A summary of the compression applied to cancer cells via different membrane thicknesses for externally applied pressures and the resulting maximum piston contact pressures is shown in Table 1. Fig 2b further shows that comparable piston contact pressures were achieved by mildly compressing cells with applied external pressures, which were experimentally matching the pressure amounts in simulations (R^2 = 0.9967). ……”

Revised to

“……. Fig 2(b) further shows that comparable piston contact pressures were achieved by mildly compressing cells with applied external pressures (R^2 = 0.9967), which were experimentally applied according to the pressure amounts in simulations Fig 2(a). …….”

7. Page 6, line 198: “maximum piston contact pressure”…the authors should include how much variation in pressure exists for the entire area under the piston, and address how this variation may affect results

New supplementary figure, S2 Fig in the revised manuscript, has been added to illustrate the distribution of contact pressure under piston. It is referenced in the manuscript text in “Computational modelling used to calculate the maximum piston contact pressure (S2 Fig) was further validated experimentally using independent microdevices with different membrane thicknesses to enhance reproducibility and repeatability of our flexible microdevice-based compression method.”

Explanation for the piston contact pressure illustrations has been added in figure caption as follows: “S2 Fig. Illustrations of the piston contact pressure area by the symmetrical half-cell of the piston and cell-culture chamber in the computational model. They show the distribution of contact pressure (in N/m^2) under piston for the selected applied external pressures (in mbar). Red dashed line of model symmetry drawn on the illustrations at different pressures point out the middle side of the piston. The contact pressure is slightly higher at the edges of the piston towards the side channel walls where the attached membrane distance between piston and side wall is shorter. Thus, there seem to be some variation due to how the piston bottom gets squashed in these sides of the piston, but it is minimal compared to the overall area. This is demonstrated by the color being relatively even over the whole area, especially for externally applied lower pressures such as 0-420 mbar.”

8. Page 8, line 234: It is not clear why authors chose 5 min pressure stage followed by 5 min rest stage. Experimental characterization of nucleus deformation recovery time in relationship to pressure AND/OR theoretical support is needed.

The cyclic stress schedule (i.e. 5-min compressed stage followed by 5-min rest stage in each cycle) was chosen to allow cells enough time during each stage to complete bulge formation or recovery, processes which were considered important for the investigation of cell deformation and recovery after compression. Membrane bulge formation and recovery processes are clearly observable in S1 Movie. This was explained in the following paragraph in section "Comparison of the experimental and computational results in micro-piston device"

"While inferring these results, the cell compression process was imaged in detail in Matrigel-coated microchannels at a speed of 1 frame per 100 ms, as shown in S1 Movie. As illustrated by this time-lapse video, the platform allowed for the temporal evolution of the dynamic cell compression to be investigated in each cycle while the piston was contacting cells and applying compression. Distinct cell membrane bulges could be observed to form during such compression at piston contact pressure of 15.6 kPa and recovery of cell membranes from these bulges was visible when pistons were lifted off during rest stages. Although the platform is capable of temporal control of cell compression (e.g. to decrease, increase or remove the pressure at any certain time interval), duration of each stage in a cycle was set to 5 minutes, corresponding to a 5-min compressed stage followed by 5-min rest stage in each cycle. This was chosen to allow cells enough time during each stage to complete bulge formation or recovery, processes which were considered important for the investigation of cell deformation and recovery after compression. Sensor readings in S1 Movie showed that the micro-piston actuated according to externally applied pressures with reliable control and could be operated in short durations gradually or suddenly when needed."

Further explanation and discussion have been also added in this section as in the following paragraph:

"Cyclic compression in the study of Ho *et al.* was applied only for 6 minutes in total [2], significantly shorter than the entire duration of a cyclic compression in this work, and results presented here suggest that cell deformation at compression stage and recovery at rest stage depends on the temporal length of the compression on cells. Particularly observable in S1 Movie, cell membrane bulge formation slowly developed during the compression stage while cells were responding to contact pressure at the interface with the PDMS piston. This was despite the fact that the piston was actuated onto the cells rapidly during cyclic compression as directed by the external pressure controller. As shown by the pressure sensor readings in the same movie, PDMS membrane and piston were consistently responding to amount, duration and mode of the applied pressure. Thus, the gradual deformation of cells and bulge formation during compression stages must originate in the response type of the cells to mechanical load when in solid contact with the PDMS piston. During rest stages, where the compression

was lifted, cell bulges did not fully recover right after compression was removed, suggesting that the cells require a certain amount of time to recover compressed cellular parts (S1 Movie). Based on these observations of temporal evolution of the cell compression application, and comparison of application durations in this study with the study by Ho *et al.* [2], we hypothesize that this recovery also depends on the duration and number of the cycles."

9. Page 9: Panels a-c in Figure 3 were not referenced in the text.

Panel a of Figure 3 has been referenced in section "Live cell actin profiles of cancer cells during sequential cyclic compression": "……., for these experiments SKOV-3 ovarian cancer cells were transduced on-chip with CellLight Actin-GFP, BacMam 2.0 baculovirus to visualize the dynamic biomechanical processes during compression (Fig 3(a))." and panels b-c referenced in "…… and actin-GFP signals of the compressed cells were captured (Fig 3(b-c))."

10. Page 11, figure 4 should include levels of significance for intended comparison.

Significance levels for intended comparison have been added onto the graph in Figure 4. Rest of all the significance test results (p-values) are in S1_File excel file. Figure 4 caption has been also updated:

"….. Student's t-test was used to determine the statistical significance of live cell actin deformation across increasing pressure ranges. Continuous horizontal bars show significant differences between compressed cell groups under micro-pistons for images taken at the time of compression at the indicated pressure ranges. Dashed horizontal bars show significant differences between the cell groups at rest under micro-piston for images taken after compression stages at the corresponding pressure ranges of the cycles. Results represent at least three independent experiments."

11. Page 9, line 295-297: It is not clear the lower actin signal from higher pressure groups is due to higher pressure or accumulated stress from the sequential testing. Independent experiments should be carried out for each pressure..

This is very good point. We have not run such independent experiments for each pressure. From the very beginning we deliberately aimed for sequential cyclic compression profiles in a dynamic manner that can mimic varying pressures in the same microenvironment that can be applied on the same sample. The platform is suitable to run individual steps of sequential cyclic compression.

We revised Fig 4 to show statistical significance levels and those were given in detail in S1 File for all groups, and further explained and discussed in text as highlighted in the revised manuscript in section "Live cell actin profiles of cancer

cells during sequential cyclic compression". Based on these, there are no significant differences at rest stages (after completion of the compression stages) until Intermediate 2 level pressures which are considered high. Thus, although cells deform throughout 1-hour cyclic compression applied at Mild pressures, loss of actin signal was not statistically significant between 1st cycle (beginning of 1-hour) and last cycle (at the end of 1-hour). Then pressure was risen to Intermediate 1 to Intermediate 2 and Severe pressures in 30 s at each increase (gradual rate) and cells were compressed for up to 2 min at each pressure level. Those are relatively gradual and short durations at those higher pressures applied sequentially, following cyclic mode mild pressure that was applied for 1 hour. Statistically significant change was seen at rest stage after Intermediate 2 level of pressures at which we think there was a mechanical impact due to high pressure. Such an impact is statistically significant again at rest stage after applied Severe pressures compared to all other levels.

"…….. Similar as to the sequence shown in Fig 1(d), first, a pressure amount ladder was formed up to a pressure in the mild compression range, represented by an externally applied pressure of 350 mbar and resulting in a piston contact pressure of 15.6 kPa, during which the piston reached the cells at the bottom surface and cells started to distinctly deform. Once this was observed, cyclic compression was applied at mild pressure for a total of 1 hour, with cells compressed for 5 min at 15.6 kPa, followed alternately by a rest for 5 min at 0 kPa. To track cell response, actin-GFP signals of the compressed cells were captured before compression, as well as during the first and last cycles of the cyclic compression stage. At the end of this sequence, the externally applied pressure was sequentially increased first to 370 mbar, then to 400 mbar and finally 640 mbar, resulting in the respective piston contact pressures of 23.8 kPa (Intermediate 1), 37.8 kPa (Intermediate 2) and 140 kPa (Severe). During this stage, cells were compressed for up to 2 min at each pressure (Fig 1(d)) and actin-GFP signals of the compressed cells were captured (Fig 3(b-c)). Throughout the application of sequential cyclic compression, cells were allowed to adjust to each next higher applied pressure. As shown in the pressure amount ladder in Fig 1(d), pressure was applied gradually in 30 s among rising steps of the ladder until the piston reached the focal plane of the cells and started cell deformation. After observing that cells were distinctly deformed at a mild compression level but not to a disruption or lysis level, compression was set to be in cyclic mode. Although the pressure was changing suddenly along the cycles as per the settings of the selected cyclic mode in the pressure controller, cells were not damaged when compressed as they resulted in being resistant at this physiological value. Next, pressure application was continued with gradual increase from 350 to 370 mbar, from 370 to 400 mbar and from 400 to 640 mbar in 30 s for each, as shown in Fig 1(d). Thus, cells could have the time to adjust during the pressure increase and show their intrinsic response to the pressurized stage of each compression level."

Further explanation and discussion after the CTCF calculations, have been also added in the next two paragraphs in text following the paragraph above in section "Live cell actin profiles of cancer cells during sequential cyclic compression".

Overall, the revised/added discussions (highlighted in the revised manuscript) include that the lower actin-GFP signal and disruption of actin cytoskeleton in these experiments indicates for cell plasticity during a cyclic compression in ovarian cancer when varying higher pressures occur in the microenvironment.

The following sentence has been also added to Conclusions: "While actin deformation at mild pressures (e.g. 15.6 kPa) in cyclic mode indicates for cell elasticity, the resultant actin disruption indicates for cell plasticity that can emerge when ovarian cancer cells are exposed to varying and higher pressures in a microenvironment."

Although we think that a mechanical compression process is dynamic inside the body and it can develop in a cumulative way (with increasing pressures over time), and thus our sequential cyclic compression can mimic such process, we also think that the reviewer is right on that actin profile investigation could be also done straight on a particular higher pressure in a discrete way, should such sudden high pressures emerge in the body.

For cell viability investigation in Onal et al 2021 Frontiers in Physics, we extensively tried on multiple devices applying compression at 640 mbar (Severe pressure), and checked for viability at initial time of the compression and after 1 hour of continuous application at 640 mbar and then on other samples we tried sequential cyclic compression up to 640 mbar with the stage of 640 mbar itself being for 2 min. At all applications, cell viability result was similar for samples exposed to 640 mbar indicating that cell response was due to the mechanical compression at this high pressure rather than its independent experiment or sequential cyclic compression (that developed with varying pressures up to 640 mbar similar to profiles that were applied in live cell actin investigation here) or the durations of the applications.

Thus, similar to those compression assays and cell viability response in Onal et al 2021 Frontiers in Physics, for high pressures we expect that actin disruptions still happens when the applications at each of Intermediate 2 or Severe level pressure ranges are done independently and actin-GFP signal is measured, in similar way as it happened in sequential cyclic compression shown in the current work.

12. Page 9, Line 304-306: Were these "resistant" cells in contact with the piston? Characterization of the interface between the piston and the cell substrate AND/OR other metrics (ie., nucleus shape/area) is needed to rule out artifact.

The resistant cells remained on the glass surface.
The relevant sentence has been slightly revised to prevent confusion: "Interestingly, a few cells on the glass surface usually remained resistant to the increasing pressure ranges, as evidenced by their retained fluorescent signal while compressed under the micro-piston."

The following clarification has been also added: "During the entire process, wide-field imaging was used and the focus was kept on one focal plane on the glass surface where cells were initially adhered to. At compressed stages, glass and PDMS piston surfaces were in contact and at the same focal plane. At the rest stages when pressure is released and piston is retracted back, to be able to gain the signal from piston surface there would be need to bring the focal plane to the suspended piston level that is on average 108 μm above the glass surface. In the region under the micro-piston, there seem to be variations in gained actin signal between the compressed and rest stages of each cycle (Fig 4), but these were not statistically significant (p>0.05, see S1 File). Thus, no significant amount of cell artifacts was attached to piston surface."

In terms of background (light artifacts), this was extracted from the images as shown in the equation in section "Imaging and data analysis". Furthermore, the calculated actin signals were compared between the region under piston and control region around the piston. Actin signal in the control regions were also compared to each other across the cycles and remained statistically insignificant (S1 File) as it was indicated in the manuscript. The following discussion has been added to the text in the same paragraph as above in Results and discussion: "Thus, the GFP-tagged actin signal in control regions was robust during the entire process, as expected [27]. This can be reference to that fluorescence changes in the region under micro-piston (i.e. decrease in actin signal) was due to the impact of mechanical compression."

13. Page 10-11: the authors should consider discussing impact of the observed disruption of actin /no recovery due to cyclic compression in ovarian cancer and the relevance to cell plasticity

The following discussion has been added in section "Live cell actin profiles of cancer cells during sequential cyclic compression": "The observed disruption of actin cytoskeleton in these experiments indicates for cell plasticity during a cyclic compression in ovarian cancer when varying higher pressures occur in the microenvironment. There is need for more studies in literature to compare our results on cell plasticity after cyclic or dynamic compression applied sequentially up to high pressures. For cell actin deformation at milder pressures, however, a distinct change in actin cytoskeleton morphology was observed by Asem *et al.* for mesothelial cells, interacting with ovarian cancer cells, under compression that is statically applied at ~3 kPa for 24 h in a bulk compression system. As a result, the interaction between ovarian cancer cells and mesothelial cells by enhanced formation of actin-based tunneling nanotubes (TNTs), and hence metastatic progression in ovarian cancer, was promoted under compression [7]."

14. Page 13, figure 5 b-d needs to include details of significance tests conducted and the significance levels detected.

Intended comparison is shown on the figures. Figure caption is revised.
"Fig 5. ……. Student's t-test was used to determine the statistical significance of nuclear deformations in compressed and recovered cells after compression."
Rest of all the significance test results (p-values) are in S2_File excel file, newly added supplementary file.
Same revisions have been applied for Figure 6 in caption and S2_File.

15. Page 12, line 352: it is not clear what specific heterogeneity the authors mean here

Heterogeneity word was clarified to prevent confusion by revising the relevant sentence into "This divergence might emerge from the variations in decrease of cell volume under compression and nuclear envelope wrinkling and ability of each cell to recover these changes after mild compression [13,28]."

16. Page 12, line 391: minute differences (?)

"Minute" word in "……minute differences in cell behaviour….." has been removed from line 391 and from the Abstract, to prevent any confusion. It was meant that the pressure change was minute (e.g. ~5 kPa difference between Fig 5 and Fig 6) but there were changes in cell response.

It was also removed from the sentence in the section "Actin cytoskeleton and nuclei profiles of cancer cells after compression and recovery": "The phenotypical response of compressed cells during recovery after compression at 20.8 kPa differed observably from that for 15.6 kPa, demonstrating the capability of the flexible microdevice to capture ~~minute~~ differences in cell behaviour after being compressed at various pressures in a physiologically relevant manner."

17. Page 13: in Fig 5(a) 15.6 kPa (o time), actin signal looks higher in compressed area compared to cells in control area? The authors should also explain the unevenness of brightness in phase contrast images

The distinct actin deformation by the increased fluorescence signals at the edges of the cells were shown on Fig 5(a) by representative arrows as it was noted on figure caption:
"Representative arrows (white) show the distinct actin deformation by the increased fluorescence signals at the edges of the cells in the compressed groups under the micro-piston at zero time after compression."

An explanation has been added in text: "Fig 5(a) shows the distinct actin deformation by the increased fluorescence signals at the edges of the cells in the compressed groups under the micro-piston at zero time after compression, compared to the control (non-compressed) groups around the micro-piston. Thus,

membrane bulges formed during cell compression at 15.6 kPa as observed in S1 Movie and fixed directly after compression appeared as increased actin signal at cell edges. Conversely, at 24 h-recovery after compression, cell actin deformation seems recovered in the group under the micro-piston as appeared similar to the control group."

A note about the unevenness of brightness in phase contrast images has been added to the caption of Fig 5(a): "The unevenness of brightness in phase contrast images is due to that imaging focal plane was on cells cultured on glass while the micro-piston, brought back to static state after compression, was suspended on average 108 μm above the glass."

18. Fig. 5 & 6 quantification of nucleus metrics: how many independent experiments were conducted?

Figure captions are revised.
"Fig 5. …… (mean ± s.e.m. n = 588, 230, 358, 174 cell nuclei from at least two or three independent experiments per each group)."
"Fig 6. …… (mean ± s.e.m. n = 406, 162, 342, 143 cell nuclei from at least two or three independent experiments per each group)."

19. Page 12, line 380: authors should explain why no significant changes in nucleus area were observed, as it would be expected given the high stress cells were experiencing. The authors should also explain the visible difference in two "0 (CR)" groups in Figure 6 (b)

All the statistical significance values for nuclear deformations in compressed and recovered cells after compression are noted in S2_File, newly added as supplementary file.

S2 File caption "Statistical significance levels among all groups, obtained with Student's t-test run for nuclear deformations in compressed and recovered cells after compression presented in Fig 5 and Fig 6." has been added. Fig 5 and Fig 6 captions have been also updated.

We added the following result and discussion for the difference between the two "0 (CR)" values: "There is statistically significant change in nuclei area between the control groups of zero-time and of 24 h-recovery after compression (see S2 File). Although these are not exactly the same control groups in the same conditions and we intend to compare the test group (e.g. zero-time or 24 h-recovery after compression) with their own control groups, we consider that the observed significant difference could be due to cell-cell signaling between the cells in compressed and control groups during that 24 h-recovery period after compression. In the groups of zero-time after compression, we did not allow

control and compressed cells enough time to further interact with each other as they were fixed right after the compression."

We further added the following discussion for the absence of statistical change in nucleus area between the control and compressed groups at zero-time after compression, in the associated paragraph: "While we expect a statistically significant change between the compressed and its control group at zero-time after compression at 20.8 kPa similar to the results observed in our previous report [5], we attribute the absence of such a significant change to cells being cultured on Matrigel-coating here as opposed to PLL-coating used in the previous experiments. Since Matrigel is expected to better represent the cancer cell microenvironment, it thus aided in cell growth into denser monolayers and formation of supporting extracellular matrix layer, that can alter sensing of the applied stress for the compressed cells."

Comments to the Editor:

This study lacks novelty.

RECOMMENDATION:

Reject.

Reviewer #3: Onal and colleagues present a previously published method to dynamically compress a monolayer of cells. In this article, they use this device to briefly study how compression can impact actin expression, through a live reporter.

The study is technically sound. However, there are some points that need to be addressed before I can reach a decision:

1/ The claim of novelty of the device needs to be taken away. The device does not seem different from the one published by the authors in 2021 in Frontiers in Physics, and is inspired by the one published by Mishra et al (PNAS 2017), which should be acknowledged. In their 2021 paper, the authors notably already perform cyclic compression. Can the authors comment on what is really new, and if nothing is, can they transform the abstract and parts of introduction / conclusion to have it more focused on the study on actin?

Multiple parts in the manuscript have been revised and novelty section has been improved as in the last paragraphs of the Introduction, as highlighted in the revised manuscript. Abstract and Conclusion have been also revised.

We thank the reviewer for letting us know about Mishra et al (PNAS 2017) which we were not aware of. We have gone through the details of the microfluidic device presented in the supplementary of the paper. The device includes 2 layers of PDMS with micro patch pad array of 15-30 μm height which can be obtained in a single master. Design and applications are more adapted to yeast-size cells. Thus, the two platforms developed by Onal et al 2021 Frontiers in Physics and Mishra et al 2017 PNAS are different in dimensions and fabrication methods. Our microfluidic platform is composed of 3 layers of PDMS with microchannels and micro-pistons of 200 – 300 μm height and we provide dynamic cell compression at mammalian cell-culture suited dimensions. The 3 layers must be fabricated separately as UV lithography of a single master with varying structures at 200-300 μm (i.e. a micro-channel of 300 mm with a suspended micro-piston of 200 μm height and also attached to a membrane of ~200 μm thick at the same time) for a fluidic layer is not feasible to pattern as in the method of Mishra et al. to obtain small structures in a patterned single fluidic layer. One of the advantages of our platform is that dimensions of any layer can be changed easily as the layer dimensions are not depending on each other by a single master. The spin coating method to obtain such large structures as monolithically attached to PDMS membrane in our platform has been also very novel to obtain structures of 200 μm height and 300 μm diameter/length micro-pistons as attached to varying membrane thicknesses (varied from 100 μm up to 345 μm) at high resolution and high fidelity on a 4-inch Si/photoresist master. Cell loading methods are also quite different between the two papers. We use piston-retracted loading, which is a dynamic method and pistons are retracted up towards control microchannel while loading cells into the bottom channel to ensure homogenous distribution of cells under and around micro-piston. About the compression values applied, Mishra et al. used 7 psi (~48 kPa) widely in their experiments and they mention that the pressure level they use is physiologically relevant, as yeast cells can experience up to about 100 psi (~690

kPa) when grown in confined geometries. These values, especially up to 690 kPa, used in yeast applications are quite high pressures compared to physiology of cancer cell compression microenvironments. Additionally, it is not clear whether 7 psi used Mishra et al. in their device is externally applied pressure or contact pressure. Since no contact pressures are mentioned in their paper (including for supplementary as well), we consider that the mentioned value is externally applied pressure and there would be need to predict what were the internal/contact pressures via more device characterization methods. In our studies we typically characterize both externally applied pressures by a pressure pump and internal/contact pressures experienced inside the microdevice as PDMS is a hyperelastic material.

2/ I was surprised that the authors present their method as a great strategy to apply cyclic compressive stresses, but did not really present results on this part (playing with frequency for instance, etc). It is discussed on p.15, but no experiments are presented. Have the authors played with this parameter? If not, maybe it would be better to put less emphasis on this in the manuscript. Also, can the authors provide a rationale for the frequency of the oscillations used in this study?

S1 Movie and S2 Movie, and Fig 1(d) representing how the sequential cyclic compression application was done for S2 Movie and Fig 3, show temporal evolution of the dynamic cell compression and a clear control of the process.

Further explanation of the profiles in Fig 1(d) has been added at the end of 1st paragraph of the section "Live cell actin profiles of cancer cells during sequential cyclic compression": "Throughout the application of sequential cyclic compression, cells were allowed to adjust to each next higher applied pressure. As shown in the pressure amount ladder in Fig 1(d), pressure was applied gradually in 30 s among rising steps of the ladder until the piston reached the focal plane of the cells and started cell deformation. After observing that cells were distinctly deformed at a mild compression level but not to a disruption or lysis level, compression was set to be in cyclic mode. Although the pressure was changing suddenly along the cycles as per the settings of the selected cyclic mode in the pressure controller, cells were not damaged when compressed as they resulted in being resistant at this physiological value. Next, pressure application was continued with gradual increase from 350 to 370 mbar, from 370 to 400 mbar and from 400 to 640 mbar in 30 s for each, as shown in Fig 1(d). Thus, cells could have the time to adjust during the pressure increase and show their intrinsic response to the pressurized stage of each compression level."

The application rates and durations of the process in S1 Movie was already shown on the moving graph in the movie itself and was also clearly noted on its caption: "Temporal evolution of the dynamic cell compression within Matrigel-coated micro-piston devices. Time-lapse live cell imaging with 100 ms per frame was recorded for ladder pressure increase from 0 kPa up to 15.6 kPa and a short cycle at 15.6 kPa with gradual increase (from 0 kPa to 15.6 kPa) and decrease (from 15.6 kPa to 0

kPa) in 30 s for each. These steps were sequentially followed by 1 h-long cyclic compression between 0 kPa and 15.6 kPa (piston contact pressure from simulation). Cells were compressed in the temporal evolution of the dynamic pressure control within Matrigel-coated micro-piston devices and dynamic changes in cells during the cyclic compression were captured in detail at a rate of 100 ms per frame."

During the mechanical characterizations of the microdevice itself, multiple profiles that the platform could apply was shown in Onal et al. 2021 Frontiers in Physics.

Thus, it was discussed and noted in this manuscript, for the frequency of the oscillations as well in the Results and discussion, section "Comparison of the experimental and computational results in micro-piston device": "Although the platform is capable of temporal control of cell compression (e.g. to decrease, increase or remove the pressure at any certain time interval), duration of each stage in a cycle was set to 5 minutes, corresponding to a 5-min compressed stage followed by 5-min rest stage in each cycle. This was chosen to allow cells enough time during each stage to complete bulge formation or recovery, processes which were considered important for the investigation of cell deformation and recovery after compression. Sensor readings in S1 Movie showed that the micro-piston actuated according to externally applied pressures with reliable control and could be operated in short durations gradually or suddenly when needed."

This was further discussed with a next paragraph added following the paragraph above in the same section: "Cyclic compression in the study of Ho *et al.* was applied only for 6 minutes in total [2], significantly shorter than the entire duration of a cyclic compression in this work, and results presented here suggest that cell deformation at compression stage and recovery at rest stage depends on the temporal length of the compression on cells. Particularly observable in S1 Movie, cell membrane bulge formation slowly developed during the compression stage while cells were responding to contact pressure at the interface with the PDMS piston. This was despite the fact that the piston was actuated onto the cells rapidly during cyclic compression as directed by the external pressure controller. As shown by the pressure sensor readings in the same movie, PDMS membrane and piston were consistently responding to amount, duration and mode of the applied pressure. Thus, the gradual deformation of cells and bulge formation during compression stages must originate in the response type of the cells to mechanical load when in solid contact with the PDMS piston. During rest stages, where the compression was lifted, cell bulges did not fully recover right after compression was removed, suggesting that the cells require a certain amount of time to recover compressed cellular parts (S1 Movie). Based on these observations of temporal evolution of the cell compression application, and comparison of application durations in this study with the study by Ho *et al.* [2], we hypothesize that this recovery also depends on the duration and number of the cycles."

And supported with the paragraph revised and moved to Introduction, while introducing the literature frequencies and this work:

“To date, dynamic compression experiments in literature include frequencies of 0.1 – 30 Hz with an applied stress of 5.1, 9.3, 12.9 and 18.7 kPa on breast cancer cells over short durations (30 – 300 s), as used by Takao *et al.*, who observed a mixed mode of apoptosis and necrosis-dominant cell death in mechanically compressed monolayers in a bulk compression platform [18]. In contrast, work by Novak *et al.* used a frequency of 0.05 Hz for cyclic compression applied at 3.9 – 6.5 kPa for 24 hours [3]. They observed an increase in proliferation capacity and decrease in apoptosis for OVCAR3 and OVSAHO ovarian cancer cells cultured in 3D hydrogel components in a bioreactor at millimeter scales. In another example, Ho *et al.* applied cyclic compression by alternating between external pressures of 10 and 15 psi (68.9 and 103.4 kPa) at 0.5 Hz for 6 minutes [2]. Using a single cell microfluidic compression platform, their application resulted in full recovery and thus no permanent plastic deformation in MCF10A normal breast epithelial cells after compression [2]. In our work we add to these examples by using the capabilities of our compression platform to apply cyclic compression with a low frequency in microfluidic settings. In particular, we utilize this functionality to provide a first investigation of the effects of cell compression, deformation and recovery after compression on SKOV-3 ovarian cancer cells. As part of this we provide evidence that the amount of the pressure and duration of the application clearly affects morphology during cell compression and recovery. While compression frequencies in an *in vivo* tumor microenvironment are not yet fully known, cyclic compression is important for maintenance of cell culture during mechanical compressions in an *in vitro* experimental setup, in particular as it enhances media flow underneath the micro-pistons.”

3/ I have some difficulties with the motivations in the introduction for the choice of ovarian cancer cells. In cancer, compressive stress is very slowly increasing, and not cyclically changing. Dynamic stress, though, can be found in the heart or during breathing, or also through the peristaltic motion during digestion. These examples, which would be great to motivate the dynamic capabilities of the device, are not presented, and instead, the focus is made on cancer. Moreover, I would not call 16 or 21 kPa as “mild” compressive stresses: these are rather important. The authors may wish to re-write their introduction if they want to introduce the device through its dynamic capabilities. But in this case, ovarian cancer cells may not be the best model (although, the authors can just say they use these cells as a model cell line for this test study).

The first two paragraphs have been revised to better reflect on the importance of studying cyclic compression, and dynamic peritoneal environment of ovarian cancer cells. 2nd paragraph give insights on the physiological values that have been in the literature and further values that should be investigated:

“While some cells experience permanent plastic deformation after a repetitive mechanical tensile loading and unloading, the impact of such repetitive compression on deformation of cells remains largely unknown [1,2]. As such, the ability to apply cyclic compression is crucial for any experimental setup aimed at the study of mechanical compression taking place in cell and tissue

microenvironments [3–5]. For instance, in ovarian cancer, cells are exposed to chronic compressive stress from different sources such as tumor growth, displacement within stromal tissue and hydrostatic pressure out of ascitic fluid in peritoneal cavity [3,6]. Ascites-induced compression exists in the peritoneal microenvironment due to the ascites volume that can reach >2L and increases ovarian cancer cell adhesion to peritoneum, shown by Asem *et al.* using *in vivo* assay [7]. This peritoneal microenvironment is continuously affected by the movements of the surrounding organs, musculoskeletal dynamics, and gravity [6]. Thus, ovarian cancer cells might experience cyclic compression profiles during chronic exposure to compression from different sources and due to anatomical location of the ovary. Mimicking such compression profiles and physiological pressure values in an *in vitro* dynamic compression system is necessary to further study impact of compressive stress in cancer.

Compressive stress is estimated to reach 18.9 kPa for human tumors and can exceed 20 kPa based on the experimental data from murine tumors, while those values can be even higher in situ with the impact of the surrounding extracellular matrix in tumor microenvironment [3,8]. Compared to other cancer types such as breast and metastatic bone niches, compression studies have been limited for ovarian cancer, including for investigating the impact of various amounts of applied pressures [6]. Pressure values existing in literature that are applied for ovarian cancer have been in a range from ~3 kPa to 6.5 kPa [3,7,9]. Ovarian cancer cell response at higher applied pressures that can occur in the body need further investigation."

When it comes to categorization as Mild compression this was introduced and used so in Onal et al, 2021, Frontiers in Physics while investigating cell viability response to a wide range of pressures in 4 categories. In those previous experiments, we showed SKOV-3 ovarian cancer cells were highly viable as on average 94% for Mild (15.6–15.9 kPa) and 77% for Intermediate 1 (23.8–26.8 kPa). Thus, we preferred to continue to use the same categorization, as it was also used in this manuscript while applying sequential cyclic compression to study dynamics of live cell actin, from Mild to Intermediate 1 to Intermediate 2 and Severe pressures.

Similarly, and actually beyond the mild and intermediate pressures we used in our study for ovarian cancer cells, Hosmane et al. (2011, Lab on a Chip) showed application of compression on single axons in the range of 0-250 kPa and categorized as mild (<55 kPa), moderate (55 - 95 kPa) and severe levels (>95 kPa).

Cyclic compression has been also applied to breast cancer cells, normal breast epithelial cells, and other ovarian cancer cell lines, as revised to Introduction:

"To date, dynamic compression experiments in literature include frequencies of 0.1 – 30 Hz with an applied stress of 5.1, 9.3, 12.9 and 18.7 kPa on breast cancer cells over short durations (30 – 300 s), as used by Takao *et al.*, who observed a mixed mode of apoptosis and necrosis-dominant cell death in mechanically compressed

monolayers in a bulk compression platform [18]. In contrast, work by Novak *et al.* used a frequency of 0.05 Hz for cyclic compression applied at 3.9 – 6.5 kPa for 24 hours [3]. They observed an increase in proliferation capacity and decrease in apoptosis for OVCAR3 and OVSAHO ovarian cancer cells cultured in 3D hydrogel components in a bioreactor at millimeter scales. In another example, Ho *et al.* applied cyclic compression by alternating between external pressures of 10 and 15 psi (68.9 and 103.4 kPa) at 0.5 Hz for 6 minutes [2]. Using a single cell microfluidic compression platform, their application resulted in full recovery and thus no permanent deformation in MCF10A normal breast epithelial cells after compression [2]. In our work we add to these examples by using the capabilities of our compression platform to apply cyclic compression with a low frequency in microfluidic settings. In particular, we utilize this functionality to provide a first investigation of the effects of cell compression, deformation and recovery after compression on SKOV-3 ovarian cancer cells. As part of this we provide evidence that the amount of the pressure and duration of the application clearly affects morphology during cell compression and recovery. While compression frequencies in an *in vivo* tumor microenvironment are not yet fully known, cyclic compression is important for maintenance of cell culture during mechanical compressions in an *in vitro* experimental setup, in particular as it enhances media flow underneath the micro-pistons."

Thus, different to bulk compression platform by Takao et al. (2019, Biology Open), bioreactor compression platform at millimeter scales by Novak et al. (2020, Cancers) and single cell microfluidic compression platform by Ho et al. (2018, Frontiers in Bioeng and Biotech), in our platform with microchannels of 200 – 300 μm height we provide dynamic cell compression at mammalian cell-culture suited dimensions that can be adapted to 2D (cell monolayers, individual cells) and 2.5 (dense cultures with hydrogel coatings, cluster of cells) and in the future work 3D (spheroids and cell-embedded hydrogels) cultures.

As the reviewer pointed out, this method can be applied to all other cell types, as it was noted in Conclusions:

"As demonstrated here with controlled micro-scale mechanical cell compression and recovery in a flexible microdevice, our comprehensive method will provide a more accurate replication of cell-physiological mechanisms to study both short- and long-term effects of compression in cellular microenvironments."

4/ The actin intensity result seems correlated to survival, oddly. Can the authors plot these two parameters alongside as a function of pressure on same plot? Can the authors speculate on the decrease observed under pressure? Finally, have the authors checked for bleaching, by changing the sequence of compression for instance?

Cell viability results for the applied pressure ranges were reminded from Onal et al, 2021, Frontiers in Physics to give an idea at what viability states the cells were at these pressure ranges while investigating live cell actin cytoskeleton under

dynamic compression: "……. the actin-GFP transduced cancer cells were dynamically stimulated and compressed with various intermediate actuation pressures in a sequential cyclic manner, namely following a sequence from Mild (15.6–15.9 kPa) to Intermediate 1 (23.8–26.8 kPa), Intermediate 2 (37.8–41.7 kPa) and Severe (127.8–140 kPa) piston contact pressures. The first two ranges of this sequence fell within physiological and upper physiological pressure values and cell viability was high (on average 94% for Mild and 77% for Intermediate 1), while Intermediate 2 and Severe levels constitute hyperphysiological values and cell viability decreased (on average 43% for Intermediate 2 and 20% for Severe) [5]."

Thus, our previous report showed mechanical stress induced cell damage and death towards higher pressures so categorized as Intermediate 2 and Severe pressures, providing the degree of the damage as per the applied pressures via viability assays conducted in detail in Onal et al 2021 Frontiers in Physics.

The calculated actin signals were compared between the region under piston and control region around the piston. Actin signal in the control regions were also compared to each other across the cycles and remained statistically insignificant (S1 File) as it was indicated in the manuscript in Results and discussion in section "Live cell actin profiles of cancer cells during sequential cyclic compression": "The change in actin signal for compressed cells under micro-piston was statistically different compared to non-compressed cells in control regions around the micro-piston at all applied compression levels ($p < 0.05$, see S1 File). Actin signal changes in the control regions on the other hand, remained statistically insignificant ($p > 0.05$)."

The following discussion has been added in the same paragraph: "Thus, the GFP tagged actin signal in control regions was robust during the entire process, as expected [27]. This can be reference to that fluorescence changes in the region under micro-piston (i.e. decrease in actin signal) was due to the impact of mechanical compression."
Thus, given that there was no statistically significant difference between the control groups throughout the application, we do not have signs indicating for photobleaching.

The following sentence was noted in the same paragraph":
"At the higher pressures of Intermediate 2 and Severe, actin of most of the cells was disrupted compared to in the initial stages of Mild compression ($p < 0.05$, see S1 File)."

The next sentence in the same paragraph has been slightly revised to:
"Interestingly, a few cells on the glass surface usually remained resistant to the increasing pressure ranges, as evidenced by their retained fluorescent signal while compressed under the micro-piston."

The following discussion as relevant to the observation sentence above has been added: "During the entire process, wide-field imaging was used and the focus was

kept on one focal plane on the glass surface where cells were initially adhered to. At compressed stages, glass and PDMS piston surfaces were in contact and at the same focal plane. At the rest stages when pressure is released and piston is retracted back, to be able to gain the signal from piston surface there would be need to bring the focal plane to the suspended piston level that is on average 108 μm above the glass surface. In the region under the micro-piston, there seem to be variations in gained actin signal between the compressed and rest stages of each cycle (Fig 4), but these were not statistically significant ($p>0.05$, see S1 File). Thus, no significant amount of cell artifacts was attached to piston surface."

Overall, for analysis of actin-GFP signal we had done CTCF calculations and have now improved the graph to show the statical significances in Fig 4 in the revised manuscript. When there was statistically significant loss in actin-GFP signal and as seen on S2 Movie, actin disruption happens and that indicates for plastic response of the cells at those higher pressures. We added a discussion in the last paragraph of section "Live cell actin profiles of cancer cells during sequential cyclic compression": "The observed disruption of actin cytoskeleton in these experiments indicates for cell plasticity during a cyclic compression in ovarian cancer when varying higher pressures occur in the microenvironment. There is need for more studies in literature to compare our results on cell plasticity after cyclic or dynamic compression applied sequentially up to high pressures. For cell actin deformation at milder pressures, however, a distinct change in actin cytoskeleton morphology was observed by Asem *et al.* for mesothelial cells, interacting with ovarian cancer cells, under compression that is statically applied at ~3 kPa for 24 h in a bulk compression system. As a result, the interaction between ovarian cancer cells and mesothelial cells by enhanced formation of actin-based tunneling nanotubes (TNTs), and hence metastatic progression in ovarian cancer, was promoted under compression [7]."

In the section Conclusions it has been also added that "While actin deformation at mild pressures (e.g. 15.6 kPa) in cyclic mode indicates for cell elasticity, the resultant actin disruption indicates for cell plasticity that can emerge when ovarian cancer cells are exposed to varying and higher pressures in a microenvironment."

Minor points:
1/The paper is too affirmative at parts (for instance: p.9, "the results illustrate how the actin cytoskeleton […] changes in response to compressive stress" is overstated as the authors show a correlation, and do not provide a clear mechanism). The authors need to be more careful on their claims.

We have gone through the entire manuscript and have done revisions and additions in multiple parts, as highlighted in the revised manuscript.
We have revised Fig 4 and better referenced the associated Fig 1(d) and S1 File in text. We have further explained the associated graphs and significance values and interpreted and discussed the results. Thus, we have extensively revised the

associated section "Live cell actin profiles of cancer cells during sequential cyclic compression" as highlighted in the revised manuscript.

2/ Could the authors put all pressure values in the same unit? We sometimes read kPa, mbar or psi (which is by its very name not a pressure unit and not in the international system of units), and a harmonization would help the reader.

We used mbar to indicate the externally applied pressures as per the settings and calibrations on the pressure pump, pressure sensor and sensor readers used while running this work. This would help towards repeatability of the work and the reader to choose the appropriate pump, sensor, reader and their settings, should they like to use similar methods. We used kPa to indicate for the piston contact pressures as conversion from pascal by N/m^2 computed on the mechanical modelling. Our piston contact pressures given in kPa were compared to the literature values on contact pressures which are also given in kPa in the associated references.

mbar and kPa are the two units that seem to be commonly used in the publications in cell compression field.

A note was added in the methods section "Device operation for sequential cyclic compression" to guide the reader on how it is a better way to read the units: "Pressure values in this work are given in mbar for externally applied pressures, as per calibrated settings of the pressure pump, pressure sensor and sensor reader, while kPa is used for the piston contact pressures, which are based on conversion from values computed using mechanical modelling." The following sentence has been also added to the end of this section for a better guidance: "Our piston contact pressures given in kPa were compared to the literature values on contact pressures which are also given in kPa in the associated references."

As for psi, typically, we do not use psi in our work. psi values mentioned in our manuscript belongs to Ho *et al.* (2021, Frontiers in Bioeng and Biotech) as externally applied pressures and we also added their conversion to kPa in parentheses. We generally intend to not change the units described in our references from the literature.

3/ Part at the end of page 8 read more like introduction and the end of page 14 / beginning of page 15 read more like a discussion. Maybe they would be better placed in these specific sections to avoid confusion.

The following part has been moved to 'Introduction' from 'Results and discussion':

"To date, dynamic compression experiments in literature include frequencies of 0.1 – 30 Hz with an applied stress of 5.1, 9.3, 12.9 and 18.7 kPa on breast cancer cells over short durations (30 – 300 s), as used by Takao *et al.*, who observed a mixed mode of apoptosis and necrosis-dominant cell death in mechanically compressed

monolayers in a bulk compression platform [15]. In contrast, work by Novak *et al.* used a frequency of 0.05 Hz for cyclic compression applied at 3.9 – 6.5 kPa for 24 hours [3]. They observed an increase in proliferation capacity and decrease in apoptosis for OVCAR3 and OVSAHO ovarian cancer cells cultured in 3D hydrogel components in a bioreactor at millimeter scales. In another example, Ho *et al.* applied cyclic compression by alternating between external pressures of 10 and 15 psi (68.9 and 103.4 kPa) at 0.5 Hz for 6 minutes [2]. Using a single cell microfluidic compression platform, their application resulted in full recovery and thus no permanent plastic deformation in MCF10A normal breast epithelial cells after compression [2]. In the following we add to these examples by using the capabilities of our micro-piston platform to apply cyclic compression with a low frequency in microfluidic settings. In particular, we utilize this functionality to provide a first investigation of the effects of cell compression, deformation and recovery after compression on SKOV-3 ovarian cancer cells. As part of this we provide evidence that the amount of the pressure, duration of the application and number of the cycles clearly affects morphology and compression in this cell type. While compression frequencies in an *in vivo* tumor microenvironment are not yet fully known, cyclic compression is important for maintenance of cell culture during mechanical compressions in an *in vitro* experimental setup, in particular as it enhances media flow underneath the micro-pistons."

Some sentences are revised to

"…….. Using a single cell microfluidic compression platform, their application resulted in full recovery and thus no permanent deformation in MCF10A normal breast epithelial cells after compression [2]. In our work we add to these examples by using the capabilities of our compression platform to apply cyclic compression with a low frequency in microfluidic settings. ……. As part of this we provide evidence that the amount of the pressure and duration of the application clearly affects morphology during cell compression and recovery."

The following part has been moved to discussion part of the section 'Comparison of the experimental and computational results in micro-piston device' from the end of 'Results and discussion' (the end of page 14 / beginning of page 15):

"Cyclic compression in the study of Ho *et al.* was applied only for 6 minutes in total, significantly shorter than the entire duration of a cyclic compression in this work, and results presented here suggest that cell recovery after compression depends on the temporal length of the compression on cells. The overall extent of recovery of compressed cells represented by cell membrane bulges, the actin cytoskeleton and measurements of shape descriptors of cell nuclei can yield further insight into the plasticity of the cancer cells. Particularly observable in S1 Movie, cell plasticity depends on the amount of pressure applied, as well as the timing. Cell bulge formation slowly developed during the compression stage while cells were responding to contact pressure at the interface with the PDMS piston. This was despite the fact that the piston was actuated onto the cells rapidly as directed by the external pressure controller. As shown by the pressure sensor

readings in the same movie, PDMS membrane and piston were consistently responding to amount, duration and mode of the applied pressure. Thus, the gradual deformation of cells and bulge formation during compression stages must originate in the response type of the cells to mechanical load when in solid contact with the PDMS piston. During rest stages, where the compression was lifted, cell bulges did not fully recover right after compression was removed, suggesting that the cells require a certain amount of time to recover compressed cellular parts (S1 Movie). Based on these observations of temporal evolution of the cell compression application, and comparison of application durations in this study with the study by Ho *et al.* [2], we hypothesize that this recovery also depends on the duration and number of the cycles."

Revised to

"Cyclic compression in the study of Ho *et al.* was applied only for 6 minutes in total [2], significantly shorter than the entire duration of a cyclic compression in this work, and results presented here suggest that cell deformation at compression stage and recovery at rest stage depends on the temporal length of the compression on cells. Particularly observable in S1 Movie, cell membrane bulge formation slowly developed during the compression stage while cells were responding to contact pressure at the interface with the PDMS piston. This was despite the fact that the piston was actuated onto the cells rapidly during cyclic compression as directed by the external pressure controller. As shown by the pressure sensor readings in the same movie, PDMS membrane and piston were consistently responding to amount, duration and mode of the applied pressure. Thus, the gradual deformation of cells and bulge formation during compression stages must originate in the response type of the cells to mechanical load when in solid contact with the PDMS piston. During rest stages, where the compression was lifted, cell bulges did not fully recover right after compression was removed, suggesting that the cells require a certain amount of time to recover compressed cellular parts (S1 Movie). Based on these observations of temporal evolution of the cell compression application, and comparison of application durations in this study with the study by Ho *et al.* [2], we hypothesize that this recovery also depends on the duration and number of the cycles."

The following sentence that was at the end of the paragraph above has been slightly revised and moved to last paragraph of "Results and discussion":

"Based on this we conclude that the presented sequential cyclic compression and cell recovery after compression method should be employed in the future to study the effects of compression and decompression in intratumor vessels or endothelial cells to help understand the structure and functionality of decompressed vessels, importance of which was shown in a study by Padera *et al.* [25]."

Revised to

“For instance, we propose that the presented sequential cyclic compression and cell recovery after compression method should be employed in the future to study the effects of compression and decompression in intratumor vessels or endothelial cells to help understand the structure and functionality of decompressed vessels, importance of which was shown in a study by Padera *et al.* [34].”

6. PLOS authors have the option to publish the peer review history of their article (what does this mean?). If published, this will include your full peer review and any attached files.

If you choose “no”, your identity will remain anonymous but your review may still be made public.

Do you want your identity to be public for this peer review? For information about this choice, including consent withdrawal, please see our Privacy Policy.

Reviewer #1: No

Reviewer #2: No

Reviewer #3: No

[NOTE: If reviewer comments were submitted as an attachment file, they will be attached to this email and accessible via the submission site. Please log into your account, locate the manuscript record, and check for the action link "View Attachments". If this link does not appear, there are no attachment files.]

While revising your submission, please upload your figure files to the Preflight Analysis and Conversion Engine (PACE) digital diagnostic tool, https://pacev2.apexcovantage.com/. PACE helps ensure that figures meet PLOS requirements. To use PACE, you must first register as a user. Registration is free. Then, login and navigate to the UPLOAD tab, where you will find detailed instructions on how to use the tool. If you encounter any issues or have any questions when using PACE, please email PLOS at figures@plos.org. Please note that Supporting Information files do not need this step.
