# Supplementary material for: Application of sequential cyclic compression on cancer cells in a flexible microdevice
Source: PLoS One. 2023 Jan 5;18(1):e0279896. doi: 10.1371/journal.pone.0279896 (PMC9815655; doi:10.1371/journal.pone.0279896)
Supplement: Attachment — Submitted filename: Response to Reviewers.pdf [file pone.0279896.s013.pdf]

Faculty of Engineering

Department of Electrical and Computer Engineering
University of Canterbury
Private Bag 4800, Christchurch
NEW ZEALAND

Tel: +64 3 369 4652

UNIVERSITY OF CANTERBURY
Te Whare Wānanga o Waitaha
CHRISTCHURCH NEW ZEALAND

November 3, 2022

The Editor
PLOS One
PLOS Publishing

DETAILED RESPONSES TO THE JOURNAL AND REVIEWERS

Reviewers' questions/comments are underlined.
Authors' responses are given in bold font.
Blue highlighted: Previous or existing parts in the previously submitted manuscript.
Green highlighted: Revised or newly added parts in the revised version of the manuscript.

Reviewers' comments:

Reviewer's Responses to Questions
Comments to the Author

1. If the authors have adequately addressed your comments raised in a previous round of review and you feel that this manuscript is now acceptable for publication, you may indicate that here to bypass the "Comments to the Author" section, enter your conflict of interest statement in the "Confidential to Editor" section, and submit your "Accept" recommendation.
Reviewer #1: (No Response)
Reviewer #2: (No Response)

---

2. Is the manuscript technically sound, and do the data support the conclusions?

The manuscript must describe a technically sound piece of scientific research with data that supports the conclusions. Experiments must have been conducted rigorously, with appropriate controls, replication, and sample sizes. The conclusions must be drawn appropriately based on the data presented.
Reviewer #1: Partly
Reviewer #2: Partly

---

3. Has the statistical analysis been performed appropriately and rigorously?
Reviewer #1: Yes
Reviewer #2: I Don't Know

---

4. Have the authors made all data underlying the findings in their manuscript fully available?

The PLOS Data policy requires authors to make all data underlying the findings described in their manuscript fully available without restriction, with rare exception (please refer to the Data Availability Statement in the manuscript PDF file). The data should be provided as part of the manuscript or its supporting information, or deposited to a public repository. For example, in addition to summary statistics, the data points behind means, medians and variance measures should be available. If there are restrictions on publicly sharing data—e.g. participant privacy or use of data from a third party—those must be specified.
Reviewer #1: Yes
Reviewer #2: Yes

---

5. Is the manuscript presented in an intelligible fashion and written in standard English?

PLOS ONE does not copyedit accepted manuscripts, so the language in submitted articles must be clear, correct, and unambiguous. Any typographical or grammatical errors should be corrected at revision, so please note any specific errors here.
Reviewer #1: Yes
Reviewer #2: Yes

---

6. Review Comments to the Author

Please use the space provided to explain your answers to the questions above. You may also include additional comments for the author, including concerns about dual publication, research ethics, or publication ethics. (Please upload your review as an attachment if it exceeds 20,000 characters)

Reviewer #1: There are still some issues with the manuscript, and I have a few follow-up comments:

Original comment 1) In the introduction, it is mentioned that "...cells are exposed to chronic compressive stress from different sources such as tumour growth, displacement within stromal tissue and hydrostatic pressure out of ascitic fluid in peritoneal cavity." This is not completely accurate, as hydrostatic pressure will not deform the cell in the same way as anisotropic compression. The forces would be isotropic for fluid pressure.

Authors' response: Asem et al. (2020) Scientific Reports, show that ascites-induced compression exist in the peritoneal microenvironment due to the ascites volume that can reach >2L and cause intraperitoneal pressure (IPP) to rise from normal values of 5 mmHg to as high as 22 mmHg (~3 kPa). Ovarian cancer cell adhesion to peritoneum increased due to the impact of ascites-induced compression, shown in in vivo assay.

Asem et al. also applied a pressure of ~3 kPa on OVCAR5 or OVCAR8 cells added atop the mesothelial surface of murine peritoneal explants ex vivo for 24 hours in a bulk compression system. As a result, they observed enhanced interaction between peritoneal mesothelial cells and cancer cells via induction of tunneling nanotubes (TNT), which later lead to metastatic ovarian cancer progression. These compression-induced TNTs are actin based and aid in trafficking of mitochondria between ovarian cancer cells and mesothelial cells.

The other consequence of the ascites-induced compression through hydrostatic pressure in the peritoneal cavity has been a more linear anisotropic alignment of peritoneal collagen fibers. Such alignment of collagen is a common indication for enhanced invasion of solid tumors.

Overall, Asem et al. have found that ascites-induced compression promotes metastatic ovarian cancer progression.

This information has been summarized and added in the first paragraph of the Introduction: "…….Ascites-induced compression exists in the peritoneal microenvironment due to the ascites volume that can reach >2L and increases ovarian cancer cell adhesion to peritoneum, shown by Asem et al. using in vivo assay [7]. This peritoneal microenvironment is continuously affected by the movements of the surrounding organs, musculoskeletal dynamics, and gravity [6]…… "

Follow-up comment: It is not physically possible for isotropic fluid pressure to "compress" a cell against the peritoneal wall. So the in vitro experiments in the cited paper (which used solid anisotropic compression, not fluid pressure) did not mimic the in vivo situation. I agree that the observed increase in cell adhesion in vivo was likely due to strain-induced changes in the collagen network in the peritoneal wall. But this is a result of the fluid pressure stretching the containing tissue and not a direct effect on the cancer cells.

Indeed, Asem et al. has done in vivo compression assays in addition to in vitro and ex vivo. We would like to share the information from Asem et al. 2020 Scientific Reports in the way it is summarized by themselves:
"The majority of women with recurrent ovarian cancer (OvCa) develop malignant ascites with volumes that can reach > 2 L. The resulting elevation in intraperitoneal pressure (IPP), from normal values of 5 mmHg to as high as 22 mmHg, causes striking changes in the loading environment in the peritoneal cavity. The effect of ascites-induced changes in IPP on OvCa progression is largely unknown. Herein we model the functional consequences of ascites-induced compression on ovarian tumor cells and components of the peritoneal microenvironment using a panel of in vitro, ex vivo and in vivo assays. Results show that OvCa cell adhesion to the peritoneum was increased under compression. Moreover, compressive loads stimulated remodeling of peritoneal mesothelial cell surface ultrastructure via induction of tunneling nanotubes (TNT). TNT mediated interaction between peritoneal mesothelial cells and OvCa cells was enhanced under compression and was accompanied by transport of mitochondria from mesothelial cells to OvCa cells. Additionally, peritoneal collagen fibers adopted a more linear anisotropic alignment under compression, a collagen signature commonly correlated with enhanced invasion in solid tumors. Collectively, these findings elucidate a new role for ascites-induced compression in promoting metastatic OvCa progression."

We also would like to share examples where compression can be induced on tumor cells via isotropic pressure, these have been discussed in an upcoming (accepted) review article by Onal et al. 2022 iScience:
"Apart from compression produced by a mechanical loading unit, such as a piston, and confinement through physical constraints, indirect restriction of the volume of multicellular spheroids (MCSs) can also be used to apply compression on cells. To achieve this, Delarue et al. cultured MCSs of various cell lines including colon carcinoma, breast cancer, and sarcoma cells and supplemented the culture medium of MCSs with dextran which is a biopolymer that does not penetrate single cells [Delarue et al. 2014 Biophysical J.]. Dextran addition into media exerted a moderate osmotic stress directly on the outermost layer of cells in spheroids. This osmotic stress was transmitted to the inner cells of spheroids as mechanical compressive stress, which in turn reduced the overall volume of the MCSs. Compressive stress applied through such volume limitation inhibited cell proliferation in tumor MCSs. A similar setup was constructed by interposing a dialysis membrane between the MCSs and media with dextran, upon which the MCSs of CT26 mouse colon carcinoma cells were mechanically compressed by stress transmitted from the dialysis membrane to inner cells via osmotic stress at the outermost layer of the cells [Montel et al. 2011 Physical Rev Letters and 2012 New J of Physics]. In either method, with or without dialysis membrane, the volume of the MCS was reduced by the applied stress. Although this osmotic effect by dextran did not originate from high osmolarity, such as produced by salts, compressive stress in these setups emerged as a network stress by an osmotic origin, which impacted the tumor growth rate and MCS volume. The resultant compressive stress was estimated to be 5 kPa or 10 kPa based on the concentration of dextran added to the culture medium. Thus, it can be said that the

compressive stress transmission on the cells was not the product of a contacting physical surface, for example a flexible solid polymer (e.g. PDMS). Furthermore, this type of compression was also statically applied in bulk in 48-well plates, unlike the controlled and dynamic manner that can be achieved in microfluidic devices."

Such a concentration change and transmission of compressive stress onto cells can happen via ascites in the peritoneal cavity based on the volume and composition of the ascitic fluid that is further discussed and referenced in the following in response to 3rd follow-up comment of the reviewer.

Original comment 3) Does the disruption of the cytoskeleton depend on the rate of application of the force? If the piston is applied more slowly, do the cells have time to adjust and avoid damage?

Authors' response: Regarding deformation and disruption of actin cytoskeleton, Figure 3 and Figure 4 show how live cell actin cytoskeleton behave under dynamic compression applied over time in cyclic mode at a physiological value and sequentially in varying applied pressures in ascending order, and are out of at least 3 independent experiments. In text, while explaining the associated figures, the representative pressure profile for sequential cyclic compression process was indicated to be in Figure 1(d): "Similar as to the sequence shown in Fig 1(d), first, a pressure amount ladder was formed up to a pressure in the mild compression range, represented by an externally applied pressure of 350 mbar and resulting in a piston contact pressure of 15.6 kPa, during which the piston reached the cells at the bottom surface and cells started to distinctly deform."

We think that this pressure application is gradual enough for cells to adjust to the varying pressures during sequential cyclic compression application. Mechanical cell damage that we see at higher pressures is intrinsic to cell response to higher amounts of applied pressure. The following explanation has been added at the end of the 1st paragraph in section "Live cell actin profiles of cancer cells during sequential cyclic compression":

"Throughout the application of sequential cyclic compression, cells were allowed to adjust to each next higher applied pressure. As shown in the pressure amount ladder in Fig 1(d), pressure was applied gradually in 30 s among rising steps of the ladder until the piston reached the focal plane of the cells and started cell deformation. After observing that cells were distinctly deformed at a mild compression level but not to a disruption or lysis level, compression was set to be in cyclic mode. Although the pressure was changing suddenly along the cycles as per the settings of the selected cyclic mode in the pressure controller, cells were not damaged when compressed as they resulted in being resistant at this physiological value. Next, pressure application was continued with gradual increase from 350 to 370 mbar, from 370 to 400 mbar and from 400 to 640 mbar in 30 s for each, as shown in Fig 1(d). Thus, cells could have the time to adjust during the pressure increase and show their intrinsic response to the pressurized stage of each compression level."

Follow-up comment: The question was whether 30s is "gradual" enough. Tumor growth would be expected to cause increases in solid stress much more slowly than this, and

cells may have sufficient time to reorganize mechanical structures to avoid damage.

Here first we would like to present some examples from the literature:

- Lee et al. 2018 LOC applied 1 hour (14 kPa air external pressure, static and 1 Hz dynamic, microfluidic) on alginate–chondrocyte constructs. They observed significant changes in chondrocyte deformability, no significant change in viability under dynamic compression and decrease in viability under static compression.
- Asem et al. 2020 Scientific Rep applied 1 hour (~3 kPa; ~22 mmHg, BioPress Flexcell, static) on murine peritoneal explants including mesothelial cells (that interacts with ovarian cancer cells). They observed significant changes in extracellular extensions including tunneling nanotubes (TNTs). It should be noted that the pressure here was static for 1 hour but the pressure amount ~3 kPa is relatively low compared to pressures applied in our study.
- Ho et al. 2018 Frontiers Bioeng Biotech applied 6-min cyclic compression alternating between external pressures of 10 and 15 psi (68.9 and 103.4 kPa) at 0.5 Hz on single MCF10A normal breast epithelial cells. They observed no significant change in cell height between before and after 6-min compression.
- Takao et al. 2019 Biology Open applied cyclic compression stress for short durations of 30-60 s at 0.1 - 1.0 kPa (smaller loading) at 1 - 30 Hz, and of 210-300 s 5.1 - 18.7 kPa (larger loading) at 30 Hz. They observed mechanical stress-induced cell death (MSICD) in breast cancer cells, with mixed mode of 5–35% apoptosis and 5–95% necrosis dominant (smaller loading), and of 0.1–4.4% apoptosis and 5–98% necrosis dominant (larger loading), respectively.
- Hosmane et al. 2011 LOC applied varying compressive loads (0-250 kPa, contact pressures) to single axons for ~5 s. They observed growth (or regrowth) of axons in mild (<55 kPa), moderate (55-95 kPa) and severe (>95 kPa) levels of mechanical injury.

Comparing to some of the literature studies that are applied in in vitro compression platforms, we think that compression rate applied as one step pressure increase per 30 s (e.g. from 300 mbar to 320 mbar in 30s) that is applied during pressure ladder is gradual enough (in the Supplementary Movie 1, the gradual response of the cells can be observed distinctly in accordance with the pressure graph). This was followed by 5 min compressed – 5 min at rest cyclic compression, and total duration which is well beyond 1 hour in sequential cyclic compression applications that were run in this work are relevant in this context as we have also seen significant differences in deformation of cellular structures before and after compression or between control (non-compressed) and compressed ovarian cancer cells (similar to that we have seen significant differences in cancer cell viability, as well, in these compression rate and durations in Onal et al. 2021 Frontiers in Physics). Indeed, the damage that we observed in cells was related more to the magnitude of the applied stress not the rate. This is presented in our results throughout the manuscript. Cells at milder applied pressures (e.g 15.6 kPa) have cellular deformations but not damage. When applied pressures were increased to Intermediate 2 (37.8–41.7 kPa) and Severe (127.8–140 kPa) piston contact pressures, cells started to get damage and those results were as expected

based on both in vitro compression devices and in vivo applied pressures reported in literature. For instance, compressive stresses up to 120 mmHg (~16 kPa) can be exerted in tumor microenvironment and would be sufficient to locally collapse tumor blood vessels in vivo which commonly happens in tumors (tumor microvascular pressures are reported to range from 6 to 17 kPa), as mentioned in study results by Helmlinger et al. 1997 Nature. Compressive stresses beyond these are also expected to happen in tumor microenvironments, and tumor cells could be resistant to those as mentioned in the Introduction of our manuscript:

"Compressive stress is estimated to reach 18.9 kPa for human tumors and can exceed 20 kPa based on the experimental data from murine tumors, while those values can be even higher in situ with the impact of the surrounding extracellular matrix in tumor microenvironment [3,8]."

Original comment 11) Further justification should be provided for the cyclic stress schedule chosen. Musculo-skeletal tissues would be expected to experience such forces, but this is not clear for ovarian cancer.

Authors' response: The cyclic stress schedule (i.e. 5-min compressed stage followed by 5-min rest stage in each cycle) was chosen to allow cells enough time during each stage to complete bulge formation or recovery, processes which were considered important for the investigation of cell deformation and recovery after compression. Membrane bulge formation and recovery processes are clearly observable in S1 Movie. This was explained in the following paragraph in section "Comparison of the experimental and computational results in micro-piston device"

"While inferring these results, the cell compression process was imaged in detail in Matrigel-coated microchannels at a speed of 1 frame per 100 ms, as shown in S1 Movie. As illustrated by this time-lapse video, the platform allowed for the temporal evolution of the dynamic cell compression to be investigated in each cycle while the piston was contacting cells and applying compression. Distinct cell membrane bulges could be observed to form during such compression at piston contact pressure of 15.6 kPa and recovery of cell membranes from these bulges was visible when pistons were lifted off during rest stages. Although the platform is capable of temporal control of cell compression (e.g. to decrease, increase or remove the pressure at any certain time interval), duration of each stage in a cycle was set to 5 minutes, corresponding to a 5-min compressed stage followed by 5-min rest stage in each cycle. This was chosen to allow cells enough time during each stage to complete bulge formation or recovery, processes which were considered important for the investigation of cell deformation and recovery after compression. Sensor readings in S1 Movie showed that the micro-piston actuated according to externally applied pressures with reliable control and could be operated in short durations gradually or suddenly when needed."

Further explanation and discussion have been also added in this section as in the following paragraph:

“Cyclic compression in the study of Ho et al. was applied only for 6 minutes in total [2], significantly shorter than the entire duration of a cyclic compression in this work, and results presented here suggest that cell deformation at compression stage and recovery at rest stage depends on the temporal length of the compression on cells. Particularly observable in S1 Movie, cell membrane bulge formation slowly developed during the compression stage while cells were responding to contact pressure at the interface with the PDMS piston. This was despite the fact that the piston was actuated onto the cells rapidly during cyclic compression as directed by the external pressure controller. As shown by the pressure sensor readings in the same movie, PDMS membrane and piston were consistently responding to amount, duration and mode of the applied pressure. Thus, the gradual deformation of cells and bulge formation during compression stages must originate in the response type of the cells to mechanical load when in solid contact with the PDMS piston. During rest stages, where the compression was lifted, cell bulges did not fully recover right after compression was removed, suggesting that the cells require a certain amount of time to recover compressed cellular parts (S1 Movie). Based on these observations of temporal evolution of the cell compression application, and comparison of application durations in this study with the study by Ho et al. [2], we hypothesize that this recovery also depends on the duration and number of the cycles.”

Regarding sources of compression ovarian cancer cells are exposed to, further information has been added in Introduction:

“While some cells experience permanent plastic deformation after a repetitive mechanical tensile loading and unloading, the impact of such repetitive compression on deformation of cells remains largely unknown [1,2]. As such, the ability to apply cyclic compression is crucial for any experimental setup aimed at the study of mechanical compression taking place in cell and tissue microenvironments [3–5]. For instance, in ovarian cancer, cells are exposed to chronic compressive stress from different sources such as tumor growth, displacement within stromal tissue and hydrostatic pressure out of ascitic fluid in peritoneal cavity [3,6]. Ascites-induced compression exists in the peritoneal microenvironment due to the ascites volume that can reach >2L and increases ovarian cancer cell adhesion to peritoneum, shown by Asem et al. using in vivo assay [7]. This peritoneal microenvironment is continuously affected by the movements of the surrounding organs, musculoskeletal dynamics, and gravity [6]. Thus, ovarian cancer cells might experience cyclic compression profiles during chronic exposure to compression from different sources and due to anatomical location of the ovary. Mimicking such compression profiles and physiological pressure values in an in vitro dynamic compression system is necessary to further study impact of compressive stress in cancer.

Compressive stress is estimated to reach 18.9 kPa for human tumors and can exceed 20 kPa based on the experimental data from murine tumors, while those values can be even higher in situ with the impact of the surrounding extracellular matrix in tumor microenvironment [3,8]. Compared to other cancer types such as breast and metastatic bone niches, compression studies have been limited for ovarian cancer, including for investigating the impact of various amounts of applied pressures [6]. Pressure values existing in literature that are applied for ovarian cancer have been in a range from ~3 kPa

to 6.5 kPa [3,7,9]. Ovarian cancer cell response at higher applied pressures that can occur in the body need further investigation."

Furthermore, it was noted in our manuscript that "While compression frequencies in an in vivo tumor microenvironment are not yet fully known, cyclic compression is important for maintenance of cell culture during mechanical compressions in an in vitro experimental setup, in particular as it enhances media flow underneath the micro-pistons."

Follow up comment: If the study is meant to investigate ovarian cancer, it should be designed to mimic the in vivo mechanical environment. The authors should either provide evidence that ovarian cancer cells are exposed to cyclic stresses in vivo, or use their device to produce different (longer term) stress application. As presented, it is not clear whether the results are an artefact of the cyclic forces.

For ovarian tumours and in progression to cancer, compression is sourced by tumour growth, so it is on inner cells in a tumour, the counteracting compression by stromal tissue and hydrostatic pressure from ascitic fluid and it is affected by the other body movements due to the anatomical region of ovarian tissue.

Although we don't have a substantial data showing cyclic compression profile in vivo in ovarian cancer patients, it would be hard to ignore the impact of the movements of the surrounding organs such as gastrointestinal movements (Rietveld, 2018, Supportive Care in Cancer), contractions and musculoskeletal dynamics that can take place in a cyclic form, which can alter the hydrostatic pressure from ascitic fluid.

When it comes to ascitic fluid and ascites-induced compression, this exists in ovarian cancer patients as it was also mentioned by Klymenko et al. 2018 Disease Models & Mechanisms, Bregenzer et al. 2019 Cancers (review article), Asem et al. 2020 Scientific reports, Novak et al. 2020 Cancers, and others.

Klymenko et al. 2018 Disease Models & Mechanisms:
"Ascites-induced increases in intraperitoneal pressure have negative implications at the clinical level. Development of tense ascites, usually associated with stage III or IV disease, is associated with severe discomfort, poor prognosis and fatality in women with EOC."
Their model systems in bulk settings, as well as the platforms by Asem et al. 2020 (bulk, ex vivo and in vivo) and Novak et al. 2020 (bioreactor at millimetre scale), have also intended to provide in vitro platforms, in comparison to microfluidic platform and method we have developed in our work, to study future mechanistic analyses of compression-induced mechanotransduction related to enhanced intraperitoneal dissemination.

We have also queried this with researchers in the Department of Obstetrics and Gynaecology, University of Otago, New Zealand, who have expertise in ovarian cancer. Here is their information they have shared with us:

“Advanced ovarian cancer has a large volume of ascitic fluid, which has high viscosity relative to water. Increased density resulting from protein components in the ascitic fluid could mimic the mechanical pressure and influence tumour cells that grow on the surface of the peritoneal membrane. The fluid could put pressure on tumour cells to change the protein cytoskeleton and reshape cell morphology and cell signalling, leading to cell survival, growth, and drug resistance.”

Klymenko et al. also mentions in their paper that ascites includes bioactive lipids, growth factors and cytokines and the role of ascites-induced changes in peritoneal mechanobiology needs to be examined.

Apart from such in vivo patient-related information about ascites-induced compression which can be impacted by the cyclic movements and pressures of the surrounding organs, that is transmitted to peritoneal microenvironment, particularly for cyclic compression profiles we insist on that this is a more realistic way of exerting compressive forces on cells whether for short or long term in an in vitro experimental setup.

Common examples for the in vitro systems that used cyclic compression at different scales:
- Takao et al. 2019 Biology Open - bulk cyclic compression device applied cyclic pressures on breast cancer cells
- Novak et al. 2020 Cancers - 3D bioreactor at millimetre scale, applied cyclic compressive forces and compared to static compression, applied on ovarian cancer models
- Ho et al. 2018 Front. Bioeng. Biotech. - microfluidic single cell compression device at micrometre scale, applied cyclic compressive forces on single normal breast epithelial cells

Briefly exemplified here, these cyclic compression applications were detailed in Introduction of our manuscript.
For more detailed information, we have introduced compressive force types and further reviewed cyclic compression profiles in our upcoming (accepted) review article by Onal et al. 2022 iScience.

Thus, unlike bulk systems which have extensive access to cell media / nutrients and a continuous compression on cells can be kept for long term which we call static compression,
when it comes to using microfluidics to study the impact of compression on cells in a microchannel (mechanical compression at micro scale), we need to do that in a cyclic manner to allow the cells to reach nutrients efficiently and so to eliminate the impact of nutrient deprivation which would happen if we had kept the compression for long term with no rest(uncompressed) cycles in between, as it was mentioned in Introduction of our manuscript:

“While compression frequencies in an in vivo tumor microenvironment are not yet fully known, cyclic compression is important for maintenance of cell culture during

mechanical compressions in an in vitro experimental setup, in particular as it enhances media flow underneath the micro-pistons.”

Reviewer #2: The authors revised the introduction and others places in the manuscript to include additional background comparing similar studies and their compression parameters with this study, and further clarifying the novelty of this study based on a previously developed device. Sufficient information is now provided to clarify Figure 2 and the corresponding results. However, some of the results require further clarification.

- Page 8, line 285: It is not clear what is the significance and relevance of cell membrane bulge formation under compression. How it is related to actin redistribution and existing literature should be discussed.

Here, the reviewer touches upon an important topic which will be focus of another piece of work. We think that cell membrane where bulge formation can happen under stress (here we expect that that bulge formation would also affect cell extensions/edges which are actin-based) and redistribution of the actin cytoskeleton would be important to investigate cell adhesion and migration(locomotion), integration of cadherin adhesion and cytoskeleton at adherens junctions at cell-cell contacts and interactions between different cell types such as ovarian cancer cells and mesothelial cells. New references (Kalli et al. 2022 Molecular Cancer Research; Mege et al. 2017 Cold Spring Harbor Perspectives in Biology; Heisenberg et al. 2013 Cell) and a comment have been added for the associated part as below:

"Changes in cell membrane integrity and redistribution of the actin cytoskeleton under stress could affect cell adhesion and migration, integration of cadherin adhesion and cytoskeleton at adherens junctions at cell-cell contacts [27–29] and interactions between different cell types such as ovarian cancer cells and mesothelial cells [7]."
Page 9, line 322-326 in the revised manuscript.

- Page 10, line 324- 359: this section is mainly describing parameters and process of the cyclic compression, therefore would better fit in Methods section.

As per the reviewer's suggestion, the relevant section "The actin-GFP transduced cancer cells were dynamically stimulated and compressed with various intermediate actuation pressures in a sequential cyclic manner,……………..……Thus, cells could have the time to adjust during the pressure increase and show their intrinsic response to the pressurized stage of each compression level." has been moved to the Methods section "Live imaging of GFP-tagged actin cytoskeleton of cells".
Page 6-7, line 207-241 in the revised manuscript.

The last sentence in the current version of the first paragraph of the Results and discussion section "Live cell actin profiles of cancer cells during sequential cyclic compression" has been slightly revised to "Specifically, the actin cytoskeleton profile was quantified while cells were in compressed and rest stages (Fig 3(b-c) and Fig 4)."
Page 10-11, line 362-363 in the revised manuscript.

- Page 10, line 333-334, "similar as to the sequence shown in fig 1(d)…": Why not use the exact sequence for generating results in fig 3 in fig 1(d) instead of a similar sequence?

Since the results in Fig 3 and Fig 4 are out of at least three independent experiments, it was meant that the sequence in Fig 1(d) is representative. It has been thus revised from "Similar as to the sequence shown in Fig 1(d)…." to "As shown by the representative sequence in Fig 1(d)….".
Page 6, line 215-216 in the revised manuscript.

- Figure 3: What is the total imaging time throughout compression? Is photobleaching of GFP a concern?

Per one sequential cyclic compression application on cell actin-GFP, as shown by the events/stages on the x-axis of the graph in Fig 4, namely from first stage 'Before compression start (0 kPa)' to the final stage 'At rest', 9 actin-GFP frames were taken. So, the total imaging itself was for 9 frames, 2 seconds exposure per frame. However, the entire of sequential cyclic compression application itself took the total time shown on the x-axis of the graph in Fig 1(d), ~8000 seconds = ~2.2 hours.

The calculated actin signals were compared between the region under piston and control region around the piston. Actin signal in the control regions were also compared to each other across the cycles and remained statistically insignificant (S1 File) as it was indicated in the manuscript in Results and discussion in section "Live cell actin profiles of cancer cells during sequential cyclic compression": "The change in actin signal for compressed cells under micro-piston was statistically different compared to non-compressed cells in control regions around the micro-piston at all applied compression levels (p <0.05, see S1 File). Actin signal changes in the control regions on the other hand, remained statistically insignificant (p >0.05)."
Page 12, line 369-373 in the revised manuscript.

The following discussion was added in the same paragraph: "Thus, the GFP tagged actin signal in control regions was robust during the entire process, as expected [30]. This can be reference to that fluorescence changes in the region under micro-piston (i.e. decrease in actin signal) was due to the impact of mechanical compression."
Page 12, line 373-376 in the revised manuscript.

Thus, given that there was no statistically significant difference between the control groups throughout the application, we do not have signs indicating for photobleaching.

- Page 11, line 411-412: The authors should be careful and more specific when describing observed changes in actin during cyclic compression. Deformation/damage

implies structural changes of actin. It is not clear whether the authors observed structural changes in actin or simply redistribution of actin inside a cell. If actin is deformed, how a lower intensity of GFP corresponds to actin deformation should be explained.

Indeed "deformation" here has meant remodelling of the actin inside a cell, similar to the term used at "nuclei deformation" or "cell deformation". When we say a cell or cellular structure is deformed, we don't necessarily mean that the cell or structure is damaged. It could be interpreted as a change in shape but not necessarily damaged shape. Compression would induce a change on cells and cellular structures, such as actin cytoskeleton and nuclei, at different levels from shape deformation to damage, depending on the strength of the applied stress. Thus, the relevant sentence has been revised as in the following:

"Thus, the cell actin was deformed and damaged only partially in the compressed part depending on the magnitude of the applied pressure, whereas it remained more intact in the non-compressed part."

Revised to

"Thus, the cell actin was either deformed or damaged in the compressed part depending on the magnitude of the applied pressure, whereas it retained more of its original shape in the non-compressed part."
Page 13, line 417-419 in the revised manuscript.

The next sentence has been also slightly revised:

"This functionality which is unique to our platform by having control and compressed groups in the same microdevice, is useful to mimic partial cellular deformation and damage that can occur in vivo in presence of a localized mechanical loading."

Revised to

"This functionality which is unique to our platform by having control and compressed groups in the same microdevice, is useful to mimic partial cellular deformation or damage that can occur in vivo in presence of a localized mechanical loading."
Page 13, line 420-422 in the revised manuscript.

- Page 11, line 376-378: Given that cell has a certain height and upon compression, part of the cell that was not in the focal plane can be compressed to the focal plane and add to the actin signal. The authors should clarify whether this was observed (maybe during one cycle of quick compression) and was an issue for the quantification. Similarly, are compressed cells and control cells in the same plane when compressed?

In the relevant sentence while we have written it as "the focus was kept on one focal plane on the glass surface where cells were initially adhered to" we did not mean exactly the glass surface itself. Before we started the sequential cyclic compression process and time-lapse imaging, we tried to have cells as many as possible in a certain focal plane (into focus) to the extent that wide-field imaging technique allows. The focal plane of cells is slightly upper than the glass surface where they are adhered to, so this was where we had the focus of most of the cells as a compromise between the very top of an adhered cell height (e.g. on top of nucleus region of a cell or a slightly more rounded cell) and the very bottom (e.g. cell extensions/edges adhered to the glass surface). Usually, unless there is a temperature change on the sample, cells stay in focus throughout the time-lapse imaging process. We have considered this and before we started the compression and imaging process, we allow the cells to come to an equilibrium in temperature on the microscope stage for 20-30 minutes.

To prevent any confusion whether the focus was on the glass surface itself or the cells themselves, the relevant sentence has been revised:

"During the entire process, wide-field imaging was used and the focus was kept on one focal plane on the glass surface where cells were initially adhered to."

Revised to

"During the entire process, wide-field imaging was used and the focus was kept on one focal plane on the cells adhered to the glass surface."
Page 12, line 380-382 in the revised manuscript.

Compressed cells and control(uncompressed) cells were in the same focal plane when compressed.

Regarding technical side of imaging cells in a dynamic compression application, an experiment by 3D imaging of the cells (taking cell height into consideration) could be useful, but also there are limitations even with the confocal imaging technique, considering how fast cells are able to react under dynamic compression and then at rest stages (once applied pressure is lifted). Related to this, we noted the following in our manuscript with a good number of references, including our own previous report, who tried confocal imaging in their compression assays and found vertical and temporal resolution and long image acquisition times required for 3D visualization are limiting factors to use confocal imaging compared to wide-field imaging:

"………. Measurement of the viscoelastic relaxation of the cells is difficult however, especially when vertical and temporal resolution in imaging are limited, and long image acquisition times required for 3D visualization might prevent capturing the changes in cell height at the time of compression. ………….. wide-field imaging is

considered fast enough (e.g. milliseconds long) to capture cell states during the steps of the compression process compared to real-time volumetric imaging (e.g. minutes long) using confocal microscopy [2,5,33–36].”
Page 16, line 520-527 in the revised manuscript.

<u>- Page 11, line 379-382: it is not clear why the focal plane needs to be at the piston level (above the glass) at rest stages, as cells were cultured on the glass slide.</u>

The section including the relevant sentence has been trying to indicate that we did not bring the focal plane to the piston level (non-compressed form, above the glass) at rest stages:
“During the entire process, wide-field imaging was used and the focus was kept on one focal plane on the glass surface where cells were initially adhered to. At compressed stages, glass and PDMS piston surfaces were in contact and at the same focal plane. At the rest stages when pressure is released and piston is retracted back, to be able to gain the signal from piston surface there would be need to bring the focal plane to the suspended piston level that is on average 108 μm above the glass surface. In the region under the micro-piston, there seem to be variations in gained actin signal between the compressed and rest stages of each cycle (Fig 4), but these were not statistically significant ($p>0.05$, see S1 File). Thus, no significant amount of cell artifacts was attached to piston surface.”

The relevant sentence has been revised and developed for further clarification:
“At the rest stages when pressure is released and piston is retracted back, to be able to gain the signal from piston surface there would be need to bring the focal plane to the suspended piston level that is on average 108 μm above the glass surface, which we did not employ in this application. Thus, we retained the same focal plane and wide-field imaging condition across all stages of the sequential compression on GFP-tagged actin of cells.”
one focal plane on the cells adhered to the glass surface.”
Page 12-13, line 383-388 in the revised manuscript.

Mentioned as response to a previous question, the first sentence has been also revised:
“During the entire process, wide-field imaging was used and the focus was kept on one focal plane on the cells adhered to the glass surface.”
Page 12, line 380-382 in the revised manuscript.

<u>- Page 11, line 386-395: Given that Ho et al used a much higher pressure compared to this study but observed actin formation instead of disruption, how much of the continuous decrease of actin signal in this study is due to insufficient rest time? Have the authors tested how long it would take cells to recover actin levels post one cycle of compression?</u>

Ho et al. 2018 Frontiers Bioeng Biotech applied 6-min cyclic compression though control valve alternating between <u>external</u> pressures of 10 and 15 psi (68.9 and

103.4 kPa) at 0.5 Hz on single MCF10A normal breast epithelial cells and observed no significant change in cell height between before and after 6-min compression. In other words, these are the pressures applied externally to operate the microfluidic device. In their study Ho et al. do not specify the actual (contact) pressure cells are experiencing inside microchannels under the blocks, which is needed for a complete comparison to our piston contact pressures and the impact on cells. 10 and 15 psi (68.9 and 103.4 kPa) Ho et al. applied externally is not what cells are experiencing as contact (internal) pressures inside microchannels. Epithelial cells would hardly stay intact/undamaged at such high pressures. Thus, compressive stress transmission onto cells must be less than their externally applied pressures.

Additionally, Ho et al. in their paper (2018 Frontiers Bioeng Biotech) mention that they have observed a limited number of cells in their single cell compression platform (in their supplementary file, there is an image of thickening of a pre-existing actin structure shown on one cell). Our study results which are an average of multiple cells out of at least three independent experiments of sequential cyclic compression application with actin-GFP are in comparison to this and they were observed for a wide range of contact (internal) pressures from Mild to Intermediate to Severe pressures (Fig 3 and 4), as it was noted in the associated part in our manuscript:

"As such, these results expand on the work by Ho et al., who used a more limited pressure and frequency range, and observed formation of new actin stress fibers and thickening of a pre-existing actin structure in a very limited number of cells [2]."
Page 13, line 396-399 in the revised manuscript.

In our study, as shown in the representative graph in Fig 1(d), there are rest stages after the compression stages were applied and we were taking the actin-GFP frame within these resting times while the cells are at rest stages. While removing the pressure from the cells (retracting the piston to its initial position) some recovery of the cells was starting. After the pressure is totally brought to zero, while there is no pressure on cells, we were waiting at least 2 minutes for the cells to recover more, and then initiating the fluorescence imaging at the wavelength and exposure setting for GFP (2 seconds per frame). We have done that consistently for the rest stages after each cycle of compression at the indicated pressure amount shown in Fig 3 and 4.

We have thought that waiting at least 2 minutes after the pressurized stage, before any GFP image was taken for the rest stage, would be somewhat enough for the cells to recover but technically it is also possible to wait more times with our sequential cyclic compression and time-lapse imaging method presented in this manuscript. The developed device and method are as flexible as it could allow for a wide range of possibilities of durations for compression and/or rest stages and for imaging.

Furthermore, in our end point staining assays of cell actin of the cyclically compressed cells at mild (15.6 kPa, for >1 hour in cyclic manner) and upper mild (20.8 kPa, for >1 hour in cyclic manner) pressures, we also see thickening of actin structures at the edges of multiple cells. We also see loss of actin structures in some cells exposed to upper mild (20.8 kPa, for >1 hour in cyclic manner) pressures as Phalloidin did not bind well to those cells while a good Hoechst signal was gained on nuclei of the same cells, shown in Figure 6(a) at zero time after compression. In the recovery process after compression, in these set of experiments, we waited 24 hours for cells to recover (keeping cells in cell culture conditions without applied pressure), after cyclic compression application was ended. The results of this are also shown in Fig 6(a) for the recovered group (at 24 h-recovery after compression).

- Page 13, line 423-426: It would be helpful to have a zoomed in image for control cells as well, so the difference of actin distribution could be observed more directly. Additionally, in Fig 5(a) compressed cells seem to have stronger actin compared to control cells? Did actin level restore in compressed cells after 24 hours or just nucleus morphology?

Zoomed in image for control cells was given in S5 Fig.
This figure has been referenced in the associated sentence:
"Fig 5(a) shows the distinct actin deformation by the increased fluorescence signals at the edges of the cells in the compressed groups under the micro-piston at zero time after compression, compared to the control (non-compressed) groups around the micro-piston (S5 Fig)."
Page 13, line 429-432 in the revised manuscript.

The quantitation demonstrated in Fig 5(b-d) is for nucleus morphology only, as nucleus has more defined shape making image analysis more convenient/standardized on a good number of cells that were interacting and forming a denser culture on Matrigel coating under a 300 mm x 300 um piston in this platform. In such a culture distinguishing actin structure for each cell would be harder and in these set of experiments cells were not transduced with actin-GFP BacMam constructs. Instead, the results were from end point staining assays, with Phalloidin staining on fixed cells at the indicated time points. For recovery of actin at a mild applied pressure amount such as 15.6 kPa, fluorescent tracking of actin of compressed cells during 24 h-recovery would give more information for quantitation of the actin structures.
However, we can say that distinct actin deformation at the edges of the cells in the compressed groups under the micro-piston at zero time after compression were no longer visible on the 24 h-recovered compressed groups in Fig 5(a). Additionally, we can say that at 24 h-recovery after compression cell actin deformation seems recovered in the group under the micro-piston as appeared similar to the control group. We mentioned these observations and slightly revised by adding in-text reference of the figures in the associated part following the sentence that has been just revised above:

“………. Thus, membrane bulges formed during cell compression at 15.6 kPa as observed in S1 Movie and fixed directly after compression appeared as increased actin signal at cell edges (Fig 5(a)). Conversely, at 24 h-recovery after compression, cell actin deformation seems recovered in the group under the micro-piston as appeared similar to the control group (Fig 5(a) and S5 Fig).”
Page 13-14, line 432-436 in the revised manuscript.

- Page 15, line 475-477: based on figure 6 it seems like the actin profile was altered after 24h recovery compared to compressed cells? It would be helpful to point out cells that did not recover in the image.

Arrows (yellow) have been added onto Figure 6.
Caption of Fig 6(a) has been revised:
“Representative arrows (white) show distinct actin deformation in form of increased fluorescence signals at the edges of the cells in the compressed groups under the micro-piston at zero time after compression.”

Revised to

“Representative white arrows show distinct actin deformation in form of increased fluorescence signals at the edges of the cells in the compressed groups under the micro-piston at zero time after compression, while yellow arrows indicate no full recovery and thus an altered actin profile of cells at 24 h-recovery after compression.”

- Page 17, line 496-498: specifying the difference, i.e., decrease/increase, would help improve the readability.

The associated sentence has been revised to improve the readability:

“On the other hand, circularity of the compressed and 24 h-recovered compressed cells was statistically different both between each and respective control groups (Fig 6(c)).”

Revised to

“On the other hand, circularity of the compressed and 24 h-recovered compressed cells statistically significantly decreased compared to their respective control groups (Fig 6(c)). Circularity of the 24 h-recovered compressed cells was also statistically less than the compressed group at zero-time after compression (Fig 6(c)).”
Page 16, line 502-505 in the revised manuscript.

7. PLOS authors have the option to publish the peer review history of their article (what does this mean?). If published, this will include your full peer review and any attached files.

If you choose “no”, your identity will remain anonymous but your review may still be made public.

Do you want your identity to be public for this peer review? For information about this choice, including consent withdrawal, please see our Privacy Policy.
Reviewer #1: No
Reviewer #2: No

[NOTE: If reviewer comments were submitted as an attachment file, they will be attached to this email and accessible via the submission site. Please log into your account, locate the manuscript record, and check for the action link "View Attachments". If this link does not appear, there are no attachment files.]

While revising your submission, please upload your figure files to the Preflight Analysis and Conversion Engine (PACE) digital diagnostic tool, https://pacev2.apexcovantage.com/. PACE helps ensure that figures meet PLOS requirements. To use PACE, you must first register as a user. Registration is free. Then, login and navigate to the UPLOAD tab, where you will find detailed instructions on how to use the tool. If you encounter any issues or have any questions when using PACE, please email PLOS at figures@plos.org. Please note that Supporting Information files do not need this step.
