# Supplementary material for: Application of sequential cyclic compression on cancer cells in a flexible microdevice
Source: PLoS One. 2023 Jan 5;18(1):e0279896. doi: 10.1371/journal.pone.0279896 (PMC9815655; doi:10.1371/journal.pone.0279896)
Supplement: Attachment — Submitted filename: Response to Reviewers.pdf [file pone.0279896.s014.pdf]

Faculty of Engineering

Department of Electrical and Computer Engineering
University of Canterbury
Private Bag 4800, Christchurch
NEW ZEALAND

Tel: +64 3 369 4652

UNIVERSITY OF CANTERBURY
Te Whare Wānanga o Waitaha
CHRISTCHURCH NEW ZEALAND

November 30, 2022

The Editor
PLOS One
PLOS Publishing

DETAILED RESPONSES TO THE JOURNAL AND REVIEWERS

Journal Requirements and Reviewers' questions/comments are underlined.
Authors' responses are given in bold font.
Blue highlighted: Previous or existing parts in the previously submitted manuscript.
Green highlighted: Revised or newly added parts in the revised version of the manuscript.

Journal Requirements:

Please review your reference list to ensure that it is complete and correct. If you have cited papers that have been retracted, please include the rationale for doing so in the manuscript text, or remove these references and replace them with relevant current references. Any changes to the reference list should be mentioned in the rebuttal letter that accompanies your revised manuscript. If you need to cite a retracted article, indicate the article's retracted status in the References list and also include a citation and full reference for the retraction notice.

We have reviewed our reference list.
Journal names in references Fabry et al. 2001 Physical Review Letters and Fu et al. 2012 Lab on a Chip have been slightly edited.
The reference Fitzpatrick M. Measuring cell fluorescence using ImageJ. The Open Lab Book; 2014 has been also improved for its BibTeX type.

Reviewers' comments:

6. Review Comments to the Author

Please use the space provided to explain your answers to the questions above. You may also include additional comments for the author, including concerns about dual publication, research ethics, or publication ethics. (Please upload your review as an attachment if it exceeds 20,000 characters)

Reviewer #1: (No Response)

Reviewer #2: I have the following comment regarding changes in GFP signal:

Page 10, line 367-368: It is still not clear what a reduction in GFP (CTCF) signal means biologically, whether it is due to changes in actin production or actin deformation, or something else.

Apart from this, the reviewer has no further comments and believes the manuscript is acceptable for publication.

The relevant sentence has been revised and developed as below:

"…… As such, the results illustrate how the actin cytoskeleton of cancer cells changes in response to compressive stress under live-cell conditions."

Revised to

"…… As such, the results illustrate how the actin cytoskeleton of cancer cells changes in response to compressive stress under live-cell conditions, from deformation to disruption, depending on the strength of compression (also see S2 Movie). The actin cytoskeleton of cells, after being flattened under the micro-piston during compression stages (Fig 3(c)), was undergoing a process of recovery during rest stages (Fig 3(b)). Throughout a cycle of compression and rest stages, actin structures rearranged themselves and a few new extensions could be observed forming, for instance, during the first cycle of applied mild pressures (15.6-15.9 kPa), while disruption and ruptures became apparent during cycles of higher pressures such as Intermediate 2 (37.8–41.7 kPa) and Severe (127.8–140 kPa) levels (see S2 Movie and Fig 3(b-c)). As such, Fig 3(b) clearly exemplifies cell actin changes from deformation to disruption over the indicated pressure ranges."
Page 10, line 367-378 in the revised manuscript.

Arrows have been added in Fig 3(b) to show cell actin changes from deformation to disruption depending on the strength of compression.
Fig 3(b) caption has been also updated as below:
"…… Yellow arrows indicate example cells exhibiting rearrangement of actin structures with formation of new extensions, blue arrows for actin deformation with

rearrangement of existing actin structures, green arrows for actin disruption with raptures and pink arrows for complete actin disruption."

Thus, in our sequential cyclic compression method, the interpretation of the reduction in GFP (CTCF) signal depends on the applied pressure range and how significant the reduction in signal is. Measuring those signals, for instance, we found that "CTCF during the rest stage after upper mild compression at Intermediate 2 level was statistically significantly different from the stage before compression ($p=0.0229$) and the rest stage after first cycle of Mild compression ($p=0.0352$), which indicates that Intermediate 2 level caused a disruption of cell actin. This disruption was significantly higher again at Severe level compared to other levels ($p <0.05$, see S1 File)", as it was indicated in text in the relevant section and in Fig 4.
The part in the following of the relevant revised/developed sentence which reviewer pointed out further detailed and discussed those results by the fluorescently measured actin signals and this part has been split into a new paragraph, starting with a small revision "In Fig 4, ……". Next paragraph provided biological interpretation comparing to other studies. A new sentence "Such a plastic response could originate from the cytoskeletal bond ruptures, as suggested by Bonakdar et al. who applied mechanical tensile loading on cells [1]." has been added in this paragraph as shown below:

"In Fig 4, the change in actin signal for compressed cells under micro-piston was statistically different compared to non-compressed cells in control regions around the micro-piston at all applied compression levels ($p <0.05$, see S1 File). Actin signal changes in the control regions on the other hand, remained statistically insignificant ($p >0.05$). Thus, the GFP-tagged actin signal in control regions was robust during the entire process, as expected [30]. This can be reference to that fluorescence changes in the region under micro-piston (i.e. decrease in actin signal) was due to the impact of mechanical compression. At the higher pressures of Intermediate 2 and Severe, actin of most of the cells was disrupted compared to in the initial stages of Mild compression ($p <0.05$, see S1 File). Interestingly, a few cells on the glass surface usually remained resistant to the increasing pressure ranges, as evidenced by their retained fluorescent signal while compressed under the micro-piston. During the entire process, wide-field imaging was used and the focus was kept on one focal plane on the cells adhered to the glass surface. At compressed stages, glass and PDMS piston surfaces were in contact and at the same focal plane. At the rest stages when pressure is released and piston is retracted back, to be able to gain the signal from piston surface there would be need to bring the focal plane to the suspended piston level that is on average 108 µm above the glass surface, which we did not employ in this application. Thus, we retained the same focal plane and wide-field imaging condition across all stages of the sequential compression on GFP-tagged actin of cells. In the region under the micro-piston, there seem to be variations in gained actin signal between the compressed and rest stages of each cycle (Fig 4), but these were not statistically significant ($p >0.05$, see S1 File). Thus, no significant amount of cell artifacts was attached to piston surface.

CTCF during the rest stage after upper mild compression at Intermediate 2 level was statistically significantly different from the stage before compression (p=0.0229) and the rest stage after first cycle of Mild compression (p=0.0352), which indicates that Intermediate 2 level caused a disruption of cell actin. This disruption was significantly higher again at Severe level compared to other levels (p <0.05, see S1 File). As such, these results expand on the work by Ho et al., who used a more limited pressure and frequency range, and observed formation of new actin stress fibers and thickening of a pre-existing actin structure in a very limited number of cells [2]. In contrast, the current work observed live-cell actin in the cytoskeleton from deformation to ruptures at a wider range of applied pressures across several stages of the sequential cyclic compression. The observed disruption of actin cytoskeleton in these experiments indicates for cell plasticity during a cyclic compression in ovarian cancer when varying higher pressures occur in the microenvironment. Such a plastic response could originate from the cytoskeletal bond ruptures, as suggested by Bonakdar et al. who applied mechanical tensile loading on cells [1]. There is need for more studies in literature to compare our results on cell plasticity after cyclic or dynamic compression applied sequentially up to high pressures. For cell actin deformation at milder pressures, however, a distinct change in actin cytoskeleton morphology was observed by Asem et al. for mesothelial cells, interacting with ovarian cancer cells, under compression that is statically applied at ~3 kPa for 24 h in a bulk compression system. As a result, the interaction between ovarian cancer cells and mesothelial cells by enhanced formation of actin-based tunneling nanotubes (TNTs), and hence metastatic progression in ovarian cancer, was promoted under compression [7].

Page 11, line 379-424 in the revised manuscript, revisions in line 379 and 414-416.

While revising your submission, please upload your figure files to the Preflight Analysis and Conversion Engine (PACE) digital diagnostic tool, https://pacev2.apexcovantage.com/. PACE helps ensure that figures meet PLOS requirements. To use PACE, you must first register as a user. Registration is free. Then, login and navigate to the UPLOAD tab, where you will find detailed instructions on how to use the tool. If you encounter any issues or have any questions when using PACE, please email PLOS at figures@plos.org. Please note that Supporting Information files do not need this step.

Fig 3 has been edited to include arrows on the images in the panel (b).
Fig 5 and Fig 6 have been slightly adjusted for matching figure dimensions.
All main figures have been checked through PACE tool. PACE corrected figure files have been uploaded to the EM submission system.
