# Supplementary material for: Application of sequential cyclic compression on cancer cells in a flexible microdevice
Source: PLoS One. 2023 Jan 5;18(1):e0279896. doi: 10.1371/journal.pone.0279896 (PMC9815655; doi:10.1371/journal.pone.0279896)
Supplement: Attachment — Submitted filename: PLOS_One_Comments.docx [file pone.0279896.s011.docx]

Reviewer Recommendation and Comments for Manuscript Number PONE-D-22-08675

Application of sequential cyclic compression on cancer cells in a flexible microdevice

Original Submission Rakesh K. Jain

Recommendation:

In this manuscript, authors characterized changes and recovery in actin fluorescence signal and nucleus shape after applying cyclic compression to ovarian cancer cells. However, there are several major issues with the current manuscript: 1) the introduction lacks information and citations of previous work and does not address the importance of this work, i.e., why apply cyclic compression on ovarian cancer cells; 2) Key parts of the experimental design lack rationale, i.e. why 5 min pressure 5 min recovery, how large is the piston/cell contact area, variability of the pressure within contact area, etc. 3) Several results are questionable, as detailed the following comments; and 4) Several results lack information on significance tests and number of independent studies. As such, the reviewer recommends rejection.

1. The introduction is not clear what the similarities and differences are between chronic and cyclic compression, and why cyclic compression is important to study in ovarian and other cancer cells.

2. Page 2, line 9-11: “Such compression is estimated to reach 18.9 kPa for human tumours and can exceed 20 kPa based on the experimental data from murine tumours [3, 7].” Given the wide range of compressive stresses in different tumor models & species, the authors should specify the physiological range of compression for ovarian cancer.

3. The introduction is not clear about the similarities and differences between chronic and cyclic compression, and why cyclic compression is important to study in ovarian and other cancer cells.
4. The innovations in the microdevice authors developed are not clear. What has been developed in the past and what is new should be summarized in the Introduction.
5. References supporting nucleus as mechanosensory are lacking. Kirby (2018) Nature Cell Biology, Fu (2012) Lab on a Chip, etc. Additionally, past findings on nucleus deformation in ovarian cancer cells and its functional effects should be introduced as background to this study
6. Page 7, Fig 2(b): it is not clear which are experimental data and which are simulated pressures on the graph, and how well they match quantitatively.
7. Page 6, line 198: “maximum piston contact pressure”…the authors should include how much variation in pressure exists for the entire area under the piston, and address how this variation may affect results
8. Page 8, line 234: It is not clear why authors chose 5 min pressure stage followed by 5 min rest stage. Experimental characterization of nucleus deformation recovery time in relationship to pressure AND/OR theoretical support is needed.
9. Page 9: Panels a-c in Figure 3 were not referenced in the text.
10. Page 11, figure 4 should include levels of significance for intended comparison.
11. Page 9, line 295-297: It is not clear the lower actin signal from higher pressure groups is due to higher pressure or accumulated stress from the sequential testing. Independent experiments should be carried out for each pressure..
12. Page 9, Line 304-306: Were these “resistant” cells in contact with the piston? Characterization of the interface between the piston and the cell substrate AND/OR other metrics (ie., nucleus shape/area) is needed to rule out artifact.
13. Page 10-11: the authors should consider discussing impact of the observed disruption of actin /no recovery due to cyclic compression in ovarian cancer and the relevance to cell plasticity
14. Page 13, figure 5 b-d needs to include details of significance tests conducted and the significance levels detected.
15. Page 12, line 352: it is not clear what specific heterogeneity the authors mean here
16. Page 12, line 391: minute differences (?)
17. Page 13: in Fig 5(a) 15.6 kPa (o time), actin signal looks higher in compressed area compared to cells in control area? The authors should also explain the unevenness of brightness in phase contrast images
18. Fig. 5 & 6 quantification of nucleus metrics: how many independent experiments were conducted?
19. Page 12, line 380: authors should explain why no significant changes in nucleus area were observed, as it would be expected given the high stress cells were experiencing. The authors should also explain the visible difference in two “0 (CR)” groups in Figure 6 (b)
